# GREM1, LRPPRC and SLC39A4 as potential biomarkers of intervertebral disc degeneration: a bioinformatics analysis based on multiple microarray and single-cell sequencing data

**DOI:** 10.1186/s12891-023-06854-4

**Published:** 2023-09-12

**Authors:** ZhaoLiang Zhang, JianZhong Huo, XingHua Ji, LinDong Wei, Jinfeng Zhang

**Affiliations:** 1 Third Hospital of Shanxi Medical University, Shanxi Bethune Hospital, Shanxi Academy of Medical Sciences, Tongji Shanxi Hospital, Taiyuan, 030032 China; 2 Taiyuan Central Hospital, Ninth Hospital of Shanxi Medical University, Southern Fendong Road 256, Taiyuan, ShanXi 030009 China

**Keywords:** Intervertebral disc degeneration, Transcriptomes, Immuno-infiltration, MicroRNAs, GREM1, SCL39A4, LRPPRC, Single-cell sequencing

## Abstract

**Background:**

Low back pain (LBP) has drawn much widespread attention and is a major global health concern. In this field, intervertebral disc degeneration (IVDD) is frequently the focus of classic studies. However, the mechanistic foundation of IVDD is unclear and has led to conflicting outcomes.

**Methods:**

Gene expression profiles (GSE34095, GSE147383) of IVDD patients alongside control groups were analyzed to identify differentially expressed genes (DEGs) in the GEO database. GSE23130 and GSE70362 were applied to validate the yielded key genes from DEGs by means of a best subset selection regression. Four machine-learning models were established to assess their predictive ability. Single-sample gene set enrichment analysis (ssGSEA) was used to profile the correlation between overall immune infiltration levels with Thompson grades and key genes. The upstream targeting miRNAs of key genes (GSE63492) were also analyzed. A single-cell transcriptome sequencing data (GSE160756) was used to define several cell clusters of nucleus pulposus (NP), annulus fibrosus (AF), and cartilaginous endplate (CEP) of human intervertebral discs and the distribution of key genes in different cell clusters was yielded.

**Results:**

By developing appropriate *p*-values and logFC values, a total of 6 DEGs was obtained. 3 key genes (LRPPRC, GREM1, and SLC39A4) were validated by an externally validated predictive modeling method. The ssGSEA results indicated that key genes were correlated with the infiltration abundance of multiple immune cells, such as dendritic cells and macrophages. Accordingly, these 4 key miRNAs (miR-103a-3p, miR-484, miR-665, miR-107) were identified as upstream regulators targeting key genes using the miRNet database and external GEO datasets. Finally, the spatial distribution of key genes in AF, CEP, and NP was plotted. Pseudo-time series and GSEA analysis indicated that the expression level of GREM1 and the differentiation trajectory of NP chondrocytes are generally consistent. GREM1 may mainly exacerbate the degeneration of NP cells in IVDD.

**Conclusions:**

Our study gives a novel perspective for identifying reliable and effective gene therapy targets in IVDD.

**Supplementary Information:**

The online version contains supplementary material available at 10.1186/s12891-023-06854-4.

## Background

Presently, the most common cause of disability in the world is low back pain (LBP). According to GBD [1] (Global Burden of Disease), around 7.8% of people worldwide experience low back pain on average each year, and this trend is accelerating as society ages and unhealthy lifestyle habits are encouraged. Intervertebral disc degeneration (IVDD) is the main cause of lower back pain at the anatomic level, and its basic lesions include fibrous tissue degeneration, disc height reduction, fibrous annulus rupture, cartilage endplate loss, annulus fibrosus mucosus, small synovial bulge formation, and disc calcification [2]. In orthopedics, IVDD is a prevalent chronic disease, whereby endoscopic and interventional treatments are among the well-developed treatment options for disc degeneration. The difficulties in determining the disease’s natural history and the dearth of population-specific diagnostic criteria and treatment alternatives have led to a consensus among academics regarding this lesion.

The basic structure of the intervertebral disc consists of the central nucleus pulposus (NP), the peripheral fibrocartilaginous annulus (AF), along with the cartilaginous endplate (CEP). Numerous studies suggest that genetic factors are another significant underlying risk factor in addition to mechanical loading, inflammatory agents, and nutritional disorders [3, 4]. The degenerative course of the intervertebral disc is thought to be a process with polygenic involvement, therefore, targeted gene therapy may account for a promising treatment regimen against IVDD in the future [4]. Recent studies suggest that immune infiltration may play a key role in the course of IVDD. The NP and AF are regarded as immune-exempt organs because of the peculiar structure of intervertebral disc tissue, which isolates them from the host’s immune system [5, 6]. The degenerated disc tissue is thought to have a specific pattern of immune cell infiltration. For example, Lan T et al. [7] analyzed the association between inflammatory response-related features and IVDD immune infiltration, revealing that IL-1β, LYN and NAMPT have the potential as biomarkers and therapeutic targets for IVDD. Silva AJ et al. [8] assessed the ways by which macrophages affected IVDD cell gene expression and provided a new therapeutic target. Deregulating the inflammatory response in the disc is a viable approach for treating IVDD, and gene therapy that targets immunological components could potentially hinder the onset of IVDD.

MicroRNAs (miRNAs) are non-coding RNAs with endogenous regulatory functions found in eukaryotes that are about 19–25 nt in length [9]. According to the competing endogenouse RNA (ceRNA) theory, miRNAs can be conjugated to their targeted mRNAs to inhibit their translation or result in mRNA degradation, thereby accomplishing the function of post-transcriptional regulation of gene expression [10]. The widespread influence of miRNA on mRNA expression and its potential value as a disease biomarker or therapeutic target has made miRNAs a significant area of basic biological and translational research, with its main application prospects focusing on disease therapy, molecular markers, and synthetic miRNAs [10–13]. Given the progress in the realm of bioinformatics and related tool-based databases, miRNA sequencing technology has grown into a powerful approach for identifying and quantitatively resolving miRNAs, circumventing the limitations of other measures. Some studies have revealed the IVDD-associated ceRNA regulatory network. Hu P et al. [14] used miRNA microarray data from the GEO database to select 9 differentially expressed microRNAs in IVDD and obtained a regulatory network containing 3 up-regulated microRNA target gene pairs and 4 down-regulated microRNA target gene pairs based on the TargetScan database. Cao S et al. [15] used AUC validation and lasso regression together with SVM screening features to construct key ceRNA regulatory networks related to oxidative stress and identified two pairs of ceRNA regulatory axes (PKD1-miR-20b-5p-AP000797 and CCNB1-miR-212-3p-AC079834). However, a common issue in related studies is the absence of the corresponding external validation and the prediction of only miRNA-mRNA pairs. Our study has predicted the target miRNAs of key mRNA sets and validated them accordingly by miRNA microarray data in GEO, leading to more credible conclusions.

The immune microenvironment in IVDD is another immune target for IVDD treatment from the perspective of immune infiltration. In conclusion, IVDD intervention and treatment based on the gene-molecular level is currently a trend in this area of research, which focuses on the ways to screen for prevalent and significant genetic markers in IVDD. In our study, this will be used as the starting point. First, the linear model was used to initially screen out the differential gene sets. External data was subsequently used to screen out the key gene sets with diagnostic values using the best subset regression method. The ssGSEA method was used to obtain related immune infiltration scores and four machine learning models were established to demonstrate the predictive ability for IVDD Thompson grades. Finally, the spatial distribution of key genes was analyzed in IVDD disc tissue using single-cell transcriptome sequencing data, which will provide reliable evidence for potential future therapeutic targets for IVDD.

## Methods

### Acquisition and preprocessing of data

The mRNA and miRNA expression data of IVDD patients were obtained from Gene Expression Omnibus (GEO) database [16] (http://www.ncbi.nlm.nih.gov/geo/), which serves as a functional public genomics database that includes high-throughput sequencing data, single-cell sequencing data, and microarray data. GSE147383 (4 young controls with 4 elderly patients) and GSE34095(3 young controls with 3 elderly patients) were used for screening and enrichment analysis of DEGs. GSE23130 (23 disc tissue samples with different Thompson grades) and GSE70362(48 disc tissue samples with different Thompson grades) were combined into one expression matrix as an external data for regression analysis, machine learning models, and ssGSEA immune infiltration analysis. GSE63492 (5 controls versus 5 patients) was subjected to microRNA expression profiling to filter out miRNAs with meaningful differential expression. GSE160756 is a single-cell sequencing data containing 3 NP,2 CEP, and 2 AF samples from human intervertebral discs using the 10X Genomics Kit, in which a total of 91,295 cells were sequenced. Pre-processing and quality control included the following steps: i) The “GEOQuery” [17] and “Bioconductor” [18] packages in R (4.2.1) were used to directly obtain the expression matrix, gene annotation files, and corresponding clinical profiles. Probe sets were converted to gene symbols according to the annotation files of the platform. Probe sets without corresponding gene symbols were removed, and the maximum expression values for different probe sets targeting the same gene were retained. ii) Boxplots were plotted for each expression matrix to recognize batch effects. Principal component analysis (PCA) and hierarchal clustering were carried out to identify intragroup differences and remove outlier samples based on the “ggplot2” package. iii) For the batch effect between samples, the “NormalizeBetweenArrays” function was used to remove them; For combined GSE23130 and GSE70362, the batch effect was eliminated using the “combat” function. Both functions were from the “limma” package [19]. iv) For GSE160756, the “Seurat” package [20] was used to perform the analysis, and the concurrent counts of the same tissue were merged using the “harmony” package [21]. According to the distribution of gene expression (at least 300 genes detected per cell) and mitochondrial gene expression (max 10%), the single-cell transcriptome sequencing data were filtered. The data were log-normalized using the “NormalizeData” function with a scale factor of 10,000 and were then normalized across all cells using the “ScaleData” function.

### Identity DEGs and functional enrichment analysis

The limma package [19] was used to construct a generalized linear model to screen the differential genes. For GSE147383 and GSE34095, the cut-off thresholds were |log2FC|> 1 and *p*-value < 0.05. Heatmaps and volcano maps of DEGs were plotted using the “ggplot2” and “pheatmap” packages; The intersection of DEGs from the two array datasets was taken to yield the final DEG set. GO terms composed of molecular functions (MFs) and biological processes (BPs) were applied based on the “clusterProfiler” package [22] of R. Statistical significance was set at *P* < 0.05.

### Definition of key genes set using a best subset selection regression

The external validation data was sourced from GSE23130 and GSE70362. The data were merged for analysis because both chips are produced by Affymetrix. The expression matrix of 72 IVDD patients containing the Thompson classification was obtained. Thompson grades I-II were defined as low degeneration grades, while grades III-V were considered as high degeneration grades as a primary outcome variable. Our subsequent analysis would be based on that data. The best subset selection for variable screening was employed due to the possibility of multicollinearity among the first screened DEGs, which could result in overfitting of the model if all of them were included in the modelingmodelling. The fundamental approach entails fitting a model to every conceivable combination of predictor variables, followed by a selection process that prioritizes the best model for each variable based on a set of standards (e.g., R2, corrected R2, MSE, Cp, AIC, SBIC, etc.), whose advantages included traversing all possible feature combinations, so the filtered features must be optimal [23, 24]. By doing so, genes with predictive value in DEGs were selected, which was defined as the Key Genes. The R packages “glmnet” [25] and “olsrr” were used in this step.

### Machine learning modeling

Using the key gene set as the independent variable, 4 supervised machine learning methods, namely, logistic regression, support vector model (SVM, with a nonlinear kernels radial basis function), randomForest (RF), and extreme gradient boosting (XGBoost), were applied to build predictive models. The combined data were randomly divided into training and validation sets at a ratio of 7:3. The training set was used to train the model, which was subsequently used for predictions using the validation set. In this model, gene expression values are used as continuous predictor variables. These supervised learning methods included nonlinear classification and linear regression. The model was established using the following R packages: “e1071”, “randomForest” and “XGBoost”. A 3-fold cross-validation was chosen to train the XGBoost classifier. The validation set data was used to assess the reliability of the model based on the area under the curve (AUC) values of the receiver operating characteristic (ROC) curve with the “pROC” package.

### In vitro experimental validation (qPCR and Western Blot)

“AF needle puncture” method was used to construct a IVDD model in rats. Sprague-Dawley rats (Female, age = 8-week-old, weight = 200–250 g) were purchased from the Animal Laboratory of Shanxi Provincial People’s Hospital. The rats were randomly divided into IVDD group (*n* = 4) and sham group (*n* = 4). The rats were anaesthetised using an intraperitoneal injection of pentobarbital (3.5 mg / 100 g). After confirming that the rats were under anaesthesia, a longitudinal incision was made in the lower abdomen in the supine position. Lumbar intervertebral discs in IVDD group rats were exposed via the posterior peritoneum and psoas. An intraoperative radiograph was used to confirm the localisation of the L5/6 disc. We used a 21 g puncture needle to penetrate parallel to the AF of the L5/6 disc. Depth of puncture is approximately 4 mm. After puncture, rotate the needle for 1 turn, hold the needle for 30 s after rotation and then pull out the needle. Rats in sham group were treated only to expose the lumbar intervertebral discs. The rats’ peritoneum, fascia and skin were sutured layer by layer. Penicillin was administered intramuscularly to the rats in both groups for 5 consecutive days after surgery (800,000 U/kg*d). At 8 weeks later, all rats were euthanised with excess carbon dioxide and lumbar spine MRI scanning. All procedures and protocols related to experimental animals were approved by the Medical Ethics Committee of Shanxi Medical University.

We used Trizol reagent (Jiangsu Biyuntian Biotechnology Institute, Nantong, China) to extract total RNA from homogenised rat intervertebral disc tissues. We used the PrimeScript™ RT Master Mix (Perfect Real Time) kit to configure the reverse transcription system (TaKaRa, Japan). We used SYBR™ Select Premixes to configure the reaction system and then performed qPCR. IVDD group and sham group were set up with 3 compound holes. (Applied Biosystems). The primers for each gene are as follows: GREM1(5ʹ- > 3ʹ): Forward primer: CGGCACTTTCCTTCGTGTTC, reverse primer: GCCGTGCGATTCATTCTGTC; LRPPRC (5ʹ- > 3ʹ): Forward primer: CAGTTAGGCACCGTGTACGA, reverse primer: GCCTCTGGTATGTCACTCGG; SLC39A4(5ʹ- > 3ʹ): Forward primer: GGGCCGTGTGAAAAGTGTCT, reverse primer: GGCGGCACTGAGGTAAGTAA; GAPDH (5ʹ- > 3ʹ): Forward primer: GGGCCGTGTGAAAAGTGTCT, reverse primer: GGCGGCACTGAGGTAAGTAA.

The rat intervertebral disc tissue samples were washed in PBS to remove blood and muscle tissue and then homogenised in the configured RIPA buffer for protein extraction. Protein concentration was measured using BCA method (Jiangsu Biyuntian Biotechnology Institute, Nantong, China). Protein blotting was performed according to standard procedures. We selected 4–12% SDS-polyacrylamide electrophoresis (SDS-PAGE) precast gel (Jiangsu Biyuntian Biotechnology Institute, Nantong, China) for separation of denatured proteins. PVDF membranes and QuickBlock™ Blocking Buffer were also purchased from Biyuntian Biotechnology Institute. Following primary antibodies were used: GREM1 Monoclonal antibody (sc-515877, Santa Cruz Biotechnology, CA), LRPPRC Polyclonal antibody (21175-1-AP, Proteintech, USA), ZIP4(SLC39A4) Polyclonal antibody (20625-1-AP, Proteintech, USA), GAPDH Polyclonal antibody (10494-1-AP, Proteintech, USA). The following secondary antibodies were used: HRP-conjugated Affinipure Goat Anti-Rabbit IgG(H + L) (SA00001-2, Proteintech, USA), HRP-conjugated Affinipure Goat Anti-Mouse IgG(H + L) (SA00001-1, Proteintech, USA). Analysis of protein bands was conducted using ImageJ software and data analysis was performed using the Prism 8 software.

### Immune infiltration analysis based on ssGSEA

A single sample enrichment analysis (ssGSEA) based on the “GSVA” package was used [26] to calculate the enrichment fraction of immune cells. Each enrichment fraction represents the extent to which genes in a particular gene set are up- or down-regulated in our external validation data. Marker information for immune cells from the article of Pornpimol Charoentong et al. [27], revealed the related genetic phenotypes of immune cell infiltration. The “pheatmap” package was used to obtain the abundance matrix of immune cells, the correlation matrix of immune cells with degenerate grades and key genes, and plot heatmaps for visual analysis.

### Targeted miRNA selection and validation

First, the target miRNAs of key genes were identified by miRNet (http://www.mirnet.ca/) database, which was developed to build miRNA–mRNA target networks. GSE63492 contains miRNA expression information of disc tissues from patients with IVDD and controls. The same method was used to screen for differentially expressed miRNAs in IVDD patients using the “limma” package. The cut-off threshold for this purpose was a* p*-value of < 0.05. Finally, the intersection of the above two miRNA sets was taken and miRNAs whose expression trends were opposite to those of their targeted mRNAs were extracted. It could be assumed that the miRNA-mRNA pairs that were predicted in this way were more reliable.

### Cellular fractionation and analysis in different tissues of IVDD

After preliminary processing, single-cell sequencing data for NP, AF, and CEP of human intervertebral discs in GSE160756 was obtained [28]. A two-step clustering method was used to cluster the cells after confirming that the batch effects within the same tissue were initially decreased using the uniform manifold approximation and projection (UMAP) method. Linear PCA clustering was followed by UMAP clustering, and the final results were visualized using UMAP plots. Cell type annotations are defined by the “SingleR” package [29]. Visualization of the space expression distribution of key genes was conducted using violin plots and UMAP plots. GSEA analysis of different cell clusters were conducted using “clusterProfiler” package. Pathways and Gene Ontology annotations were retrieved from Molecular Signature database (MSigdb). The “monocle2” package was used to analyze cell lineage relationships.

**Fig. 1:**
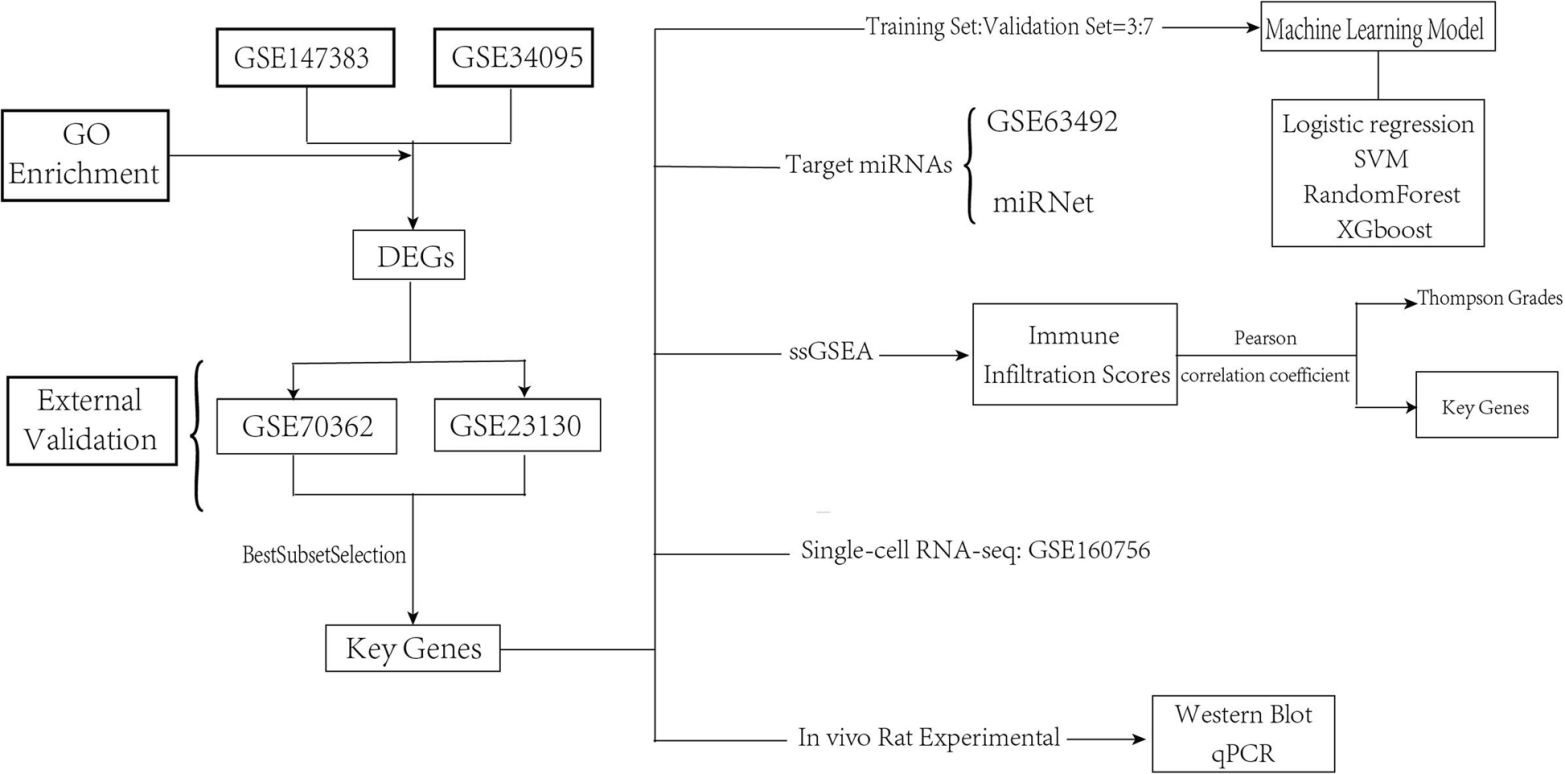
Flowchart

The flow chart for our study is listed as follows (Fig. 1).

## Results

### Screening of DEGs

**Fig. 2:**
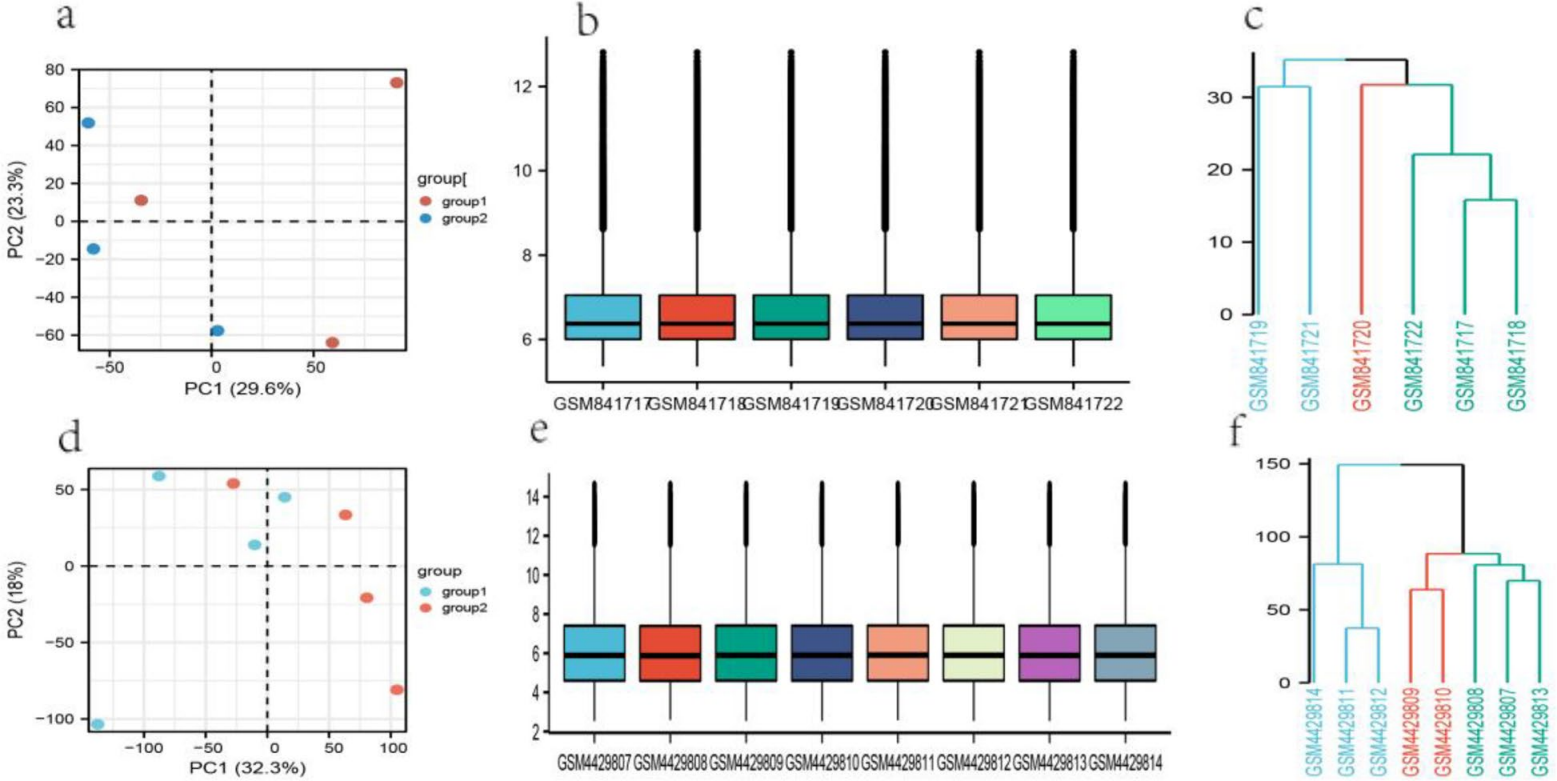
Data quality control for GSE34095 (**a**, **b**, **c**) and GSE147383 (**d**, **e**, **f**): **a**, **d** PCA plot **b**, **e** boxplot. **c**, **f** Hierarchical clustering by complete-linkage

**Fig. 3:**
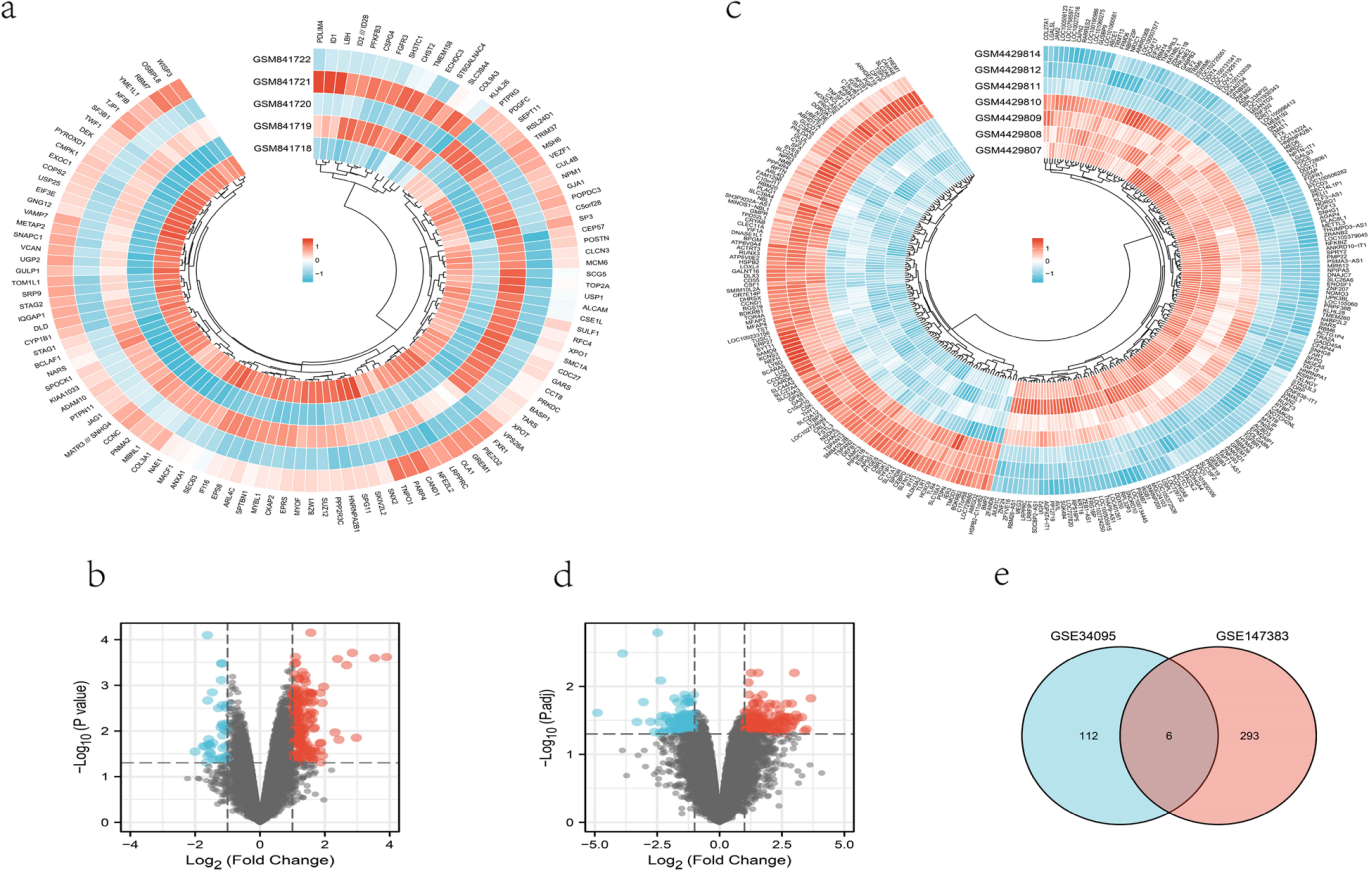
DEGs for GSE34095 (**a**, **b**) and GSE147383 (**c**, **d**) where blue represents down-regulated and red represents up-regulated genes: **a**, **c** Heatmap **b**, **d** Volcano map. Venn diagram showing the intersecting DEGs from GSE147383 and GSE34095 (**e**)

Based on the results of PCA and hierarchical clustering (Fig. 2), the following samples were discarded: GSM841717 in GSE34095 and GSM4429813 in GSE147383. The boxplot results show an essentially uniform distribution within the normalized expression matrix (Fig. 2). For GSE147383, 299 differentially expressed genes relative to normal controls were screened, of which 173 were up-regulated and 126 were down-regulated (Fig. 3). As for GSE34095, 119 differentially expressed genes were screened, of which 103 were up-regulated and 15 were down-regulated (Fig. 3). Then the intersection of both the DEGs and 6 common genes taken (LRPPRC, GREM1, XPO1, HNRNPA2B1, UGP2, SLC39A4) were used in the following analysis. LRPPRC, GREM1, XPO1, HNRNPA2B1 and UGP2 were up-regulated while SLC39A4 was down-regulated.

### Functional enrichment of both DEGs

**Fig. 4:**
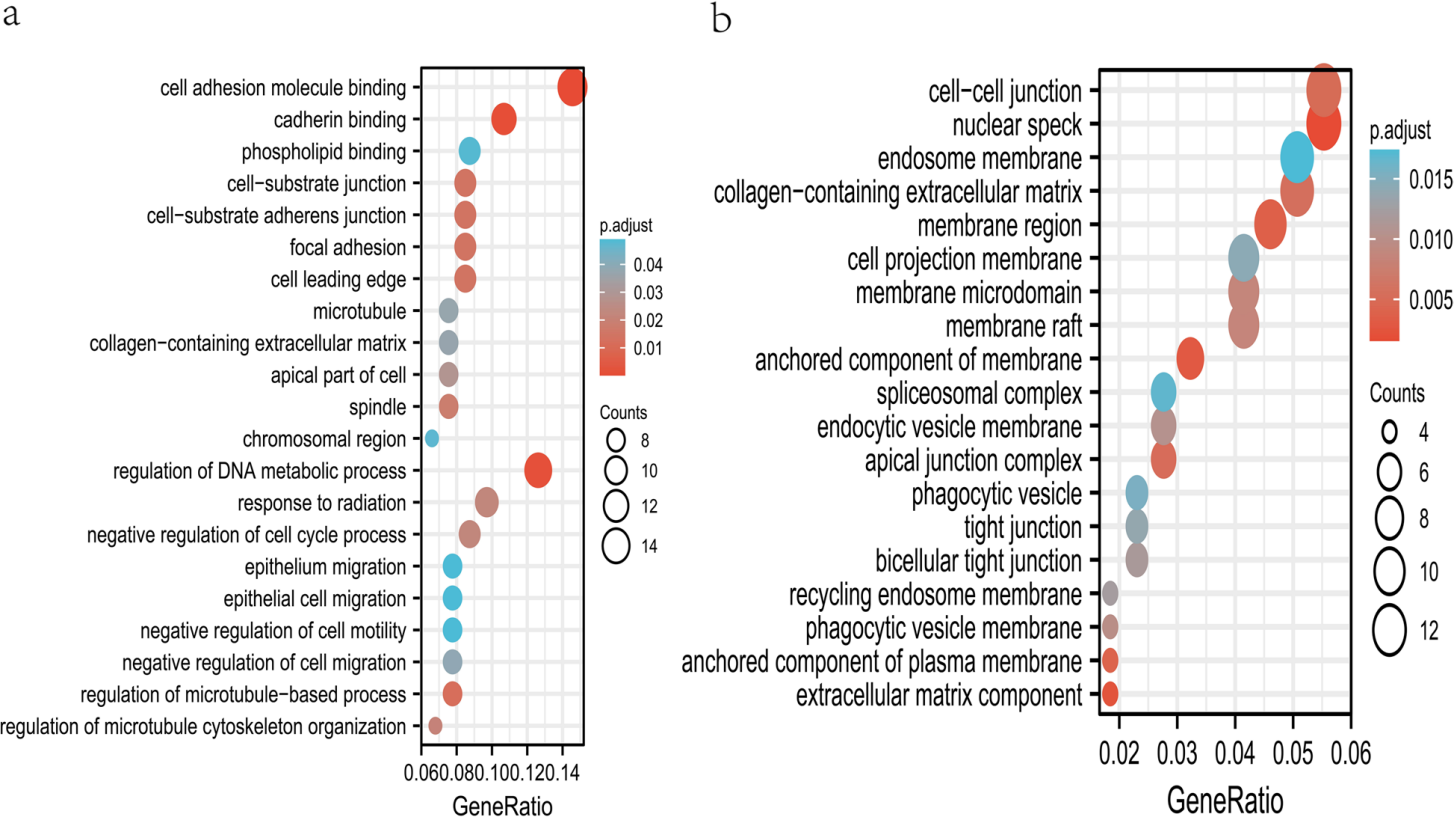
Go term enrichment results in GSE34095 (**a**) and GSE147383 (**b**)

GO enrichment analysis of DEGs in GSE34095 and GSE147383 was carried out. The GO terms whose *p-*value < 0.05 were selected and arranged in descending order according to gene counts. The terms in the DEGs were listed separately and visualized using bubble plots (Fig. 4). 3 terms in common were found: GO:0062023, GO:0008278 and GO:0010008 which were on behalf of collagen-containing extracellular matrix, cohesin complex and endosome membrane.

### Results of key genes and corresponding external validation

**Fig. 5:**
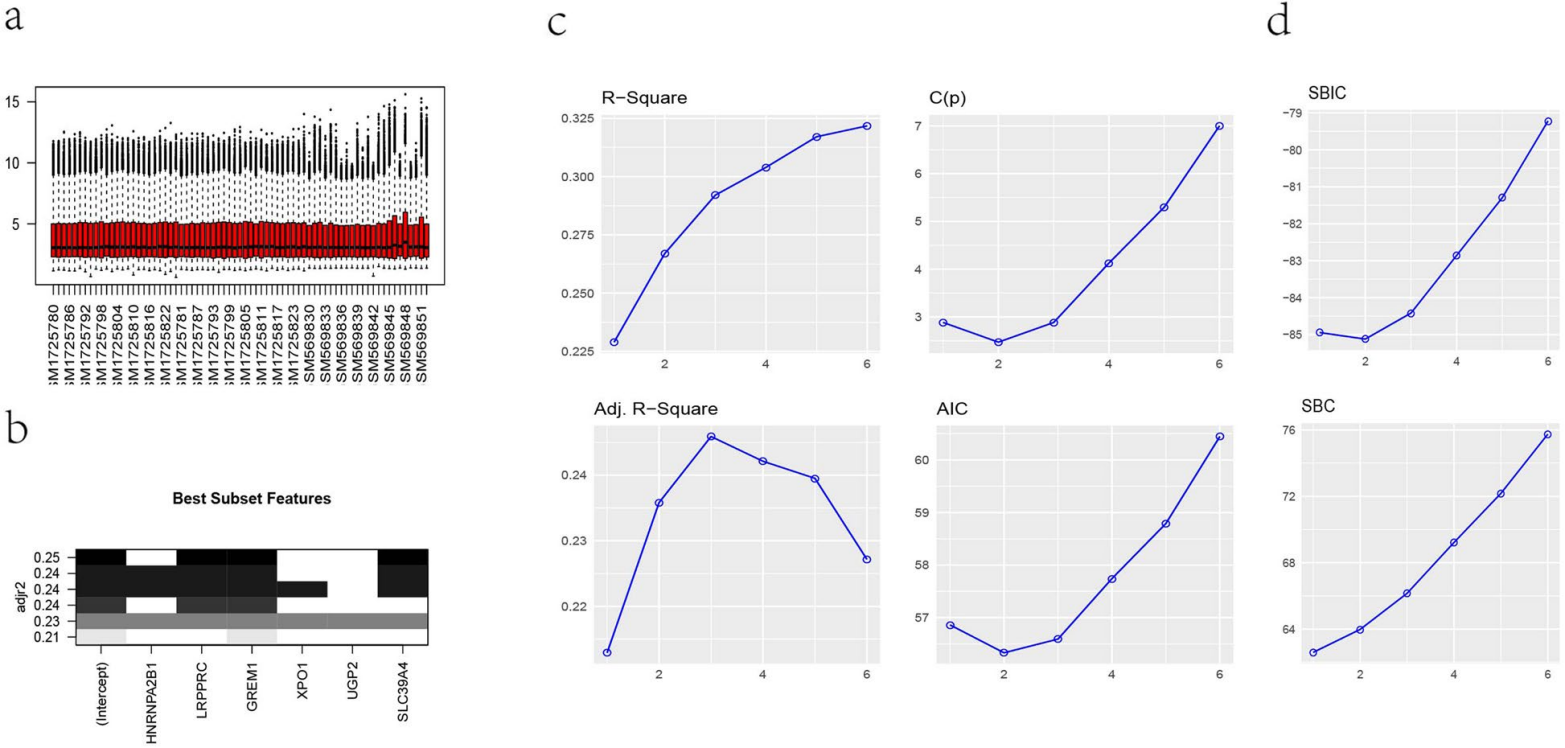
Boxplot of combined GSE147383 and GSE70362 after processing with the “combat” function (**a**). Results of the best subset selection regression: **b** The model that incorporates LRPPRC, GREM1 and SLC39A4 has the highest adjusted R square value. **c**, **d** The effect of the number of variables on the performance of each model

The dataset combined by GSE23130 and GSE70362 was regarded as independent external expression data for further analysis. The boxplot showed that the batch effects were mostly moved (Fig. 5a). The 6 obtained DEGs were incorporated into the best subset regression model, and the correlation indexes of the accuracy and fit of the model with different numbers of variables were obtained (Fig. 5b, c and d). It could be concluded that the model fit best when 3 variables were included, and was relatively accurate. Finally, LRPPRC, GREM1 and SLC39A4 were selected as key genes.

**Fig. 6:**
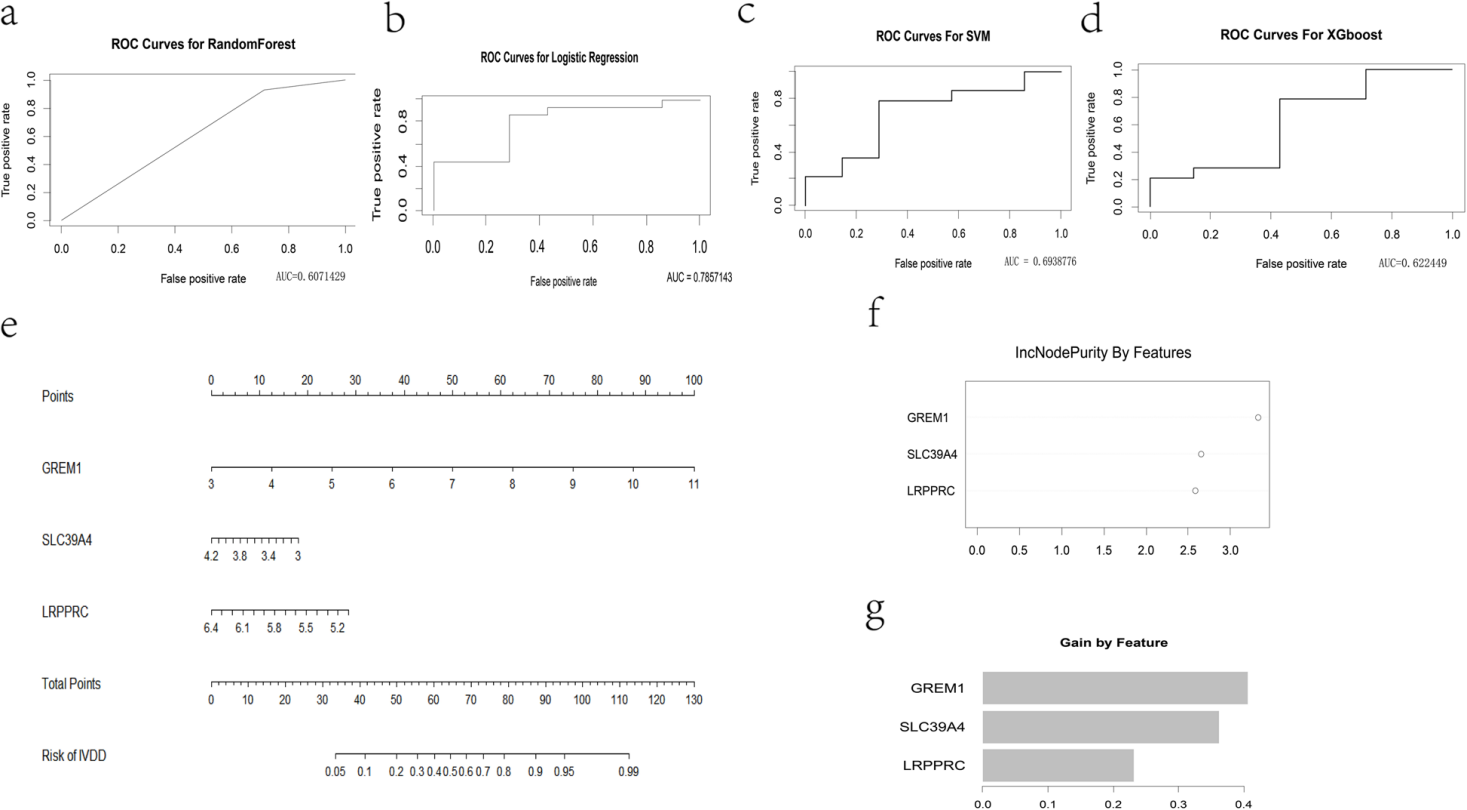
ROC plots and according AUC values in the validation set for: RandomForest (**a**), Logistic regression (**b**), SVM (**c**), XGboost (**d**). Visualization of related models: Nomogram for logistic regression (**e**), incnodepurity (**f**) and gain value (**g**) contrast for Randomforest and XGboost

The predictive capability of key genes is evaluated by 4 types of supervised machine learning methods. The ROC plots showed different results in the validation set when using different classifiers (Fig. 6). The AUC value of the logistic regression classifier reached 0.7857143 while the same for SVM, RandomForest and XGboost were 0.6938776, 0.6071429 and 0.622449 respectively, which showed that key genes had some predictive power for the defined group divided by Thompson grades in either linear or non-linear models. The nomogram of logistics was plotted and the incnodepurity and gain values in RandomForest and XGboost, were ranked which could be considered proportional to the significance of the variable (Fig. 6). It could be inferred that GREM1 had the greatest weight among all models.

### qPCR and Western Blot results

**Fig. 7:**
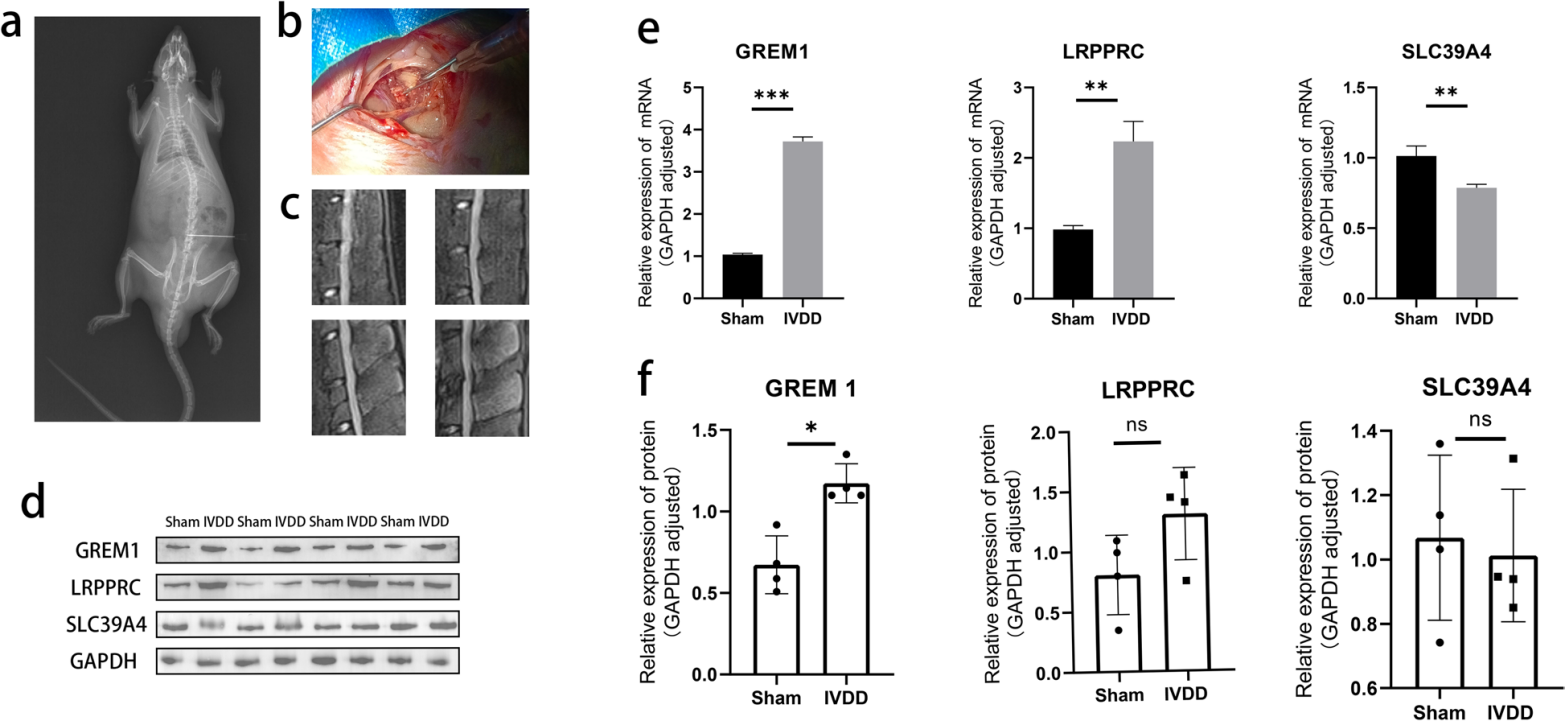
Experimental results of IVDD/Sham rats: **a** Subxiphoid localisation of rat spinal segments before surgery. **b** Puncture of intervertebral discs of anaesthetised rats in IVDD group using a 21G puncture needle. **c** MRI of the rats in IVDD group at 8 weeks after surgery showed varying degrees of reduction in disc signals and intervertebral space heights in the target segments. **e** Comparison of relative expression content of target mRNAs in Sham and IVDD groups. **d**, **f** Protein blotting bands and quantification of target proteins from Sham and IVDD groups

We performed a preliminary validation of the 3 key genes at the RNA level and protein level based on animal experiments (Fig. 7a, b). MRI scanning showed degenerative changes in the discs of the target segments of the rats in the IVDD group after 8 weeks (Fig. 7c). As expected, qPCR results showed that GREM1, LRPPRC expression were significantly up-regulated in the intervertebral discs of rats in IVDD group, while the expression level of SLC39A4 was significantly down-regulated (Fig. 7e). As complementary analyzes, Western Blots and quantitative analyses likewise confirmed part of our view that GREM1 protein was likewise more abundant in IVDD group (Fig. 7d, f). Although the expression trends of LRPPRC protein and SLC39A4 protein were similarly in line with our predictions, the differences were not statistically significant (Fig. 7f). Full-length blots are presented in Supplementary Material 4.

### Screening for targeting miRNAs

**Fig. 8:**
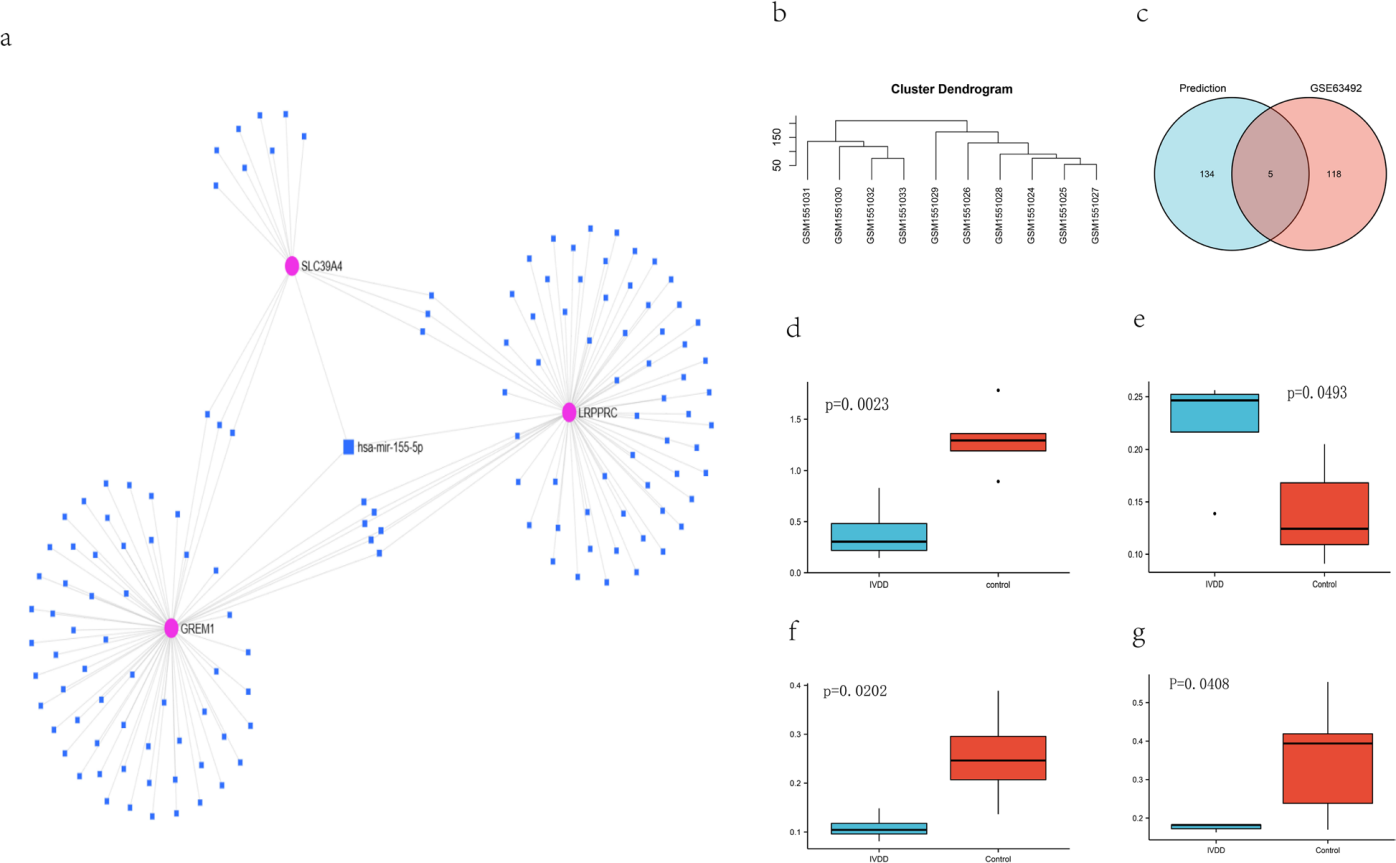
**a** Predicted target miRNAs for key genes by miRNet database including 139 miRNAs and 153 nodes. **b** Hierarchical clustering for GSE63492, indicating that GSM1551029 are identified in opposite groups. **c** The venn diagram of GSE63492 and results from database, showing 7 items in common. Differential expression of miR-665 (**d**), miR-484 (**e**), miR-107 (**f**) and miR-103a-3p (**g**) in IVDD and control groups

An interplaying miRNA-mRNA network was built using the miRNet database regarding LRPPRC, GREM1 and SLC39A4 including 139 miRNAs and 153 nodes (Fig. 8a). The same method as screening for DEGs was followed to handle with GSE63492. GSM1551029 in GSE63492 was deleted because they were identified as outlier samples in the hierarchical clustering (Fig. 8b). 124 miRNAs with significantly different expressions (*p*-value < 0.05) were identified. 7 common items were obtained by comparing the result predicted by miRNet with the miRNAs obtained from the dataset (Fig. 8c). mRNA-miRNA pairs with expression trends opposite to the target mRNA or with multiple opposite targets were selected in the degenerate disc tissues and found statistically significant differences were noted in the down-regulated expression of miR-665 (*p*-value = 0.0023) and miR-107 (*p*-value = 0.0202) in degenerating discs comparing compared to normal tissues (Fig. 8d, f), which were considered to be targets for GREM1 and LRPPRC. Interestingly, an additional fact was ascertained: For LRPPRC and SLC39A4 whose expression trends were opposite, miR-484 (*p*-value = 0.0493) and miR-103a-3p (*p*-value = 0.0408) were thought to be regulatory to both genes (Fig. 8e, g). These 4 miRNAs may have critical modulatory roles for key genes.

### ssGSEA immune infiltration results

**Fig. 9:**
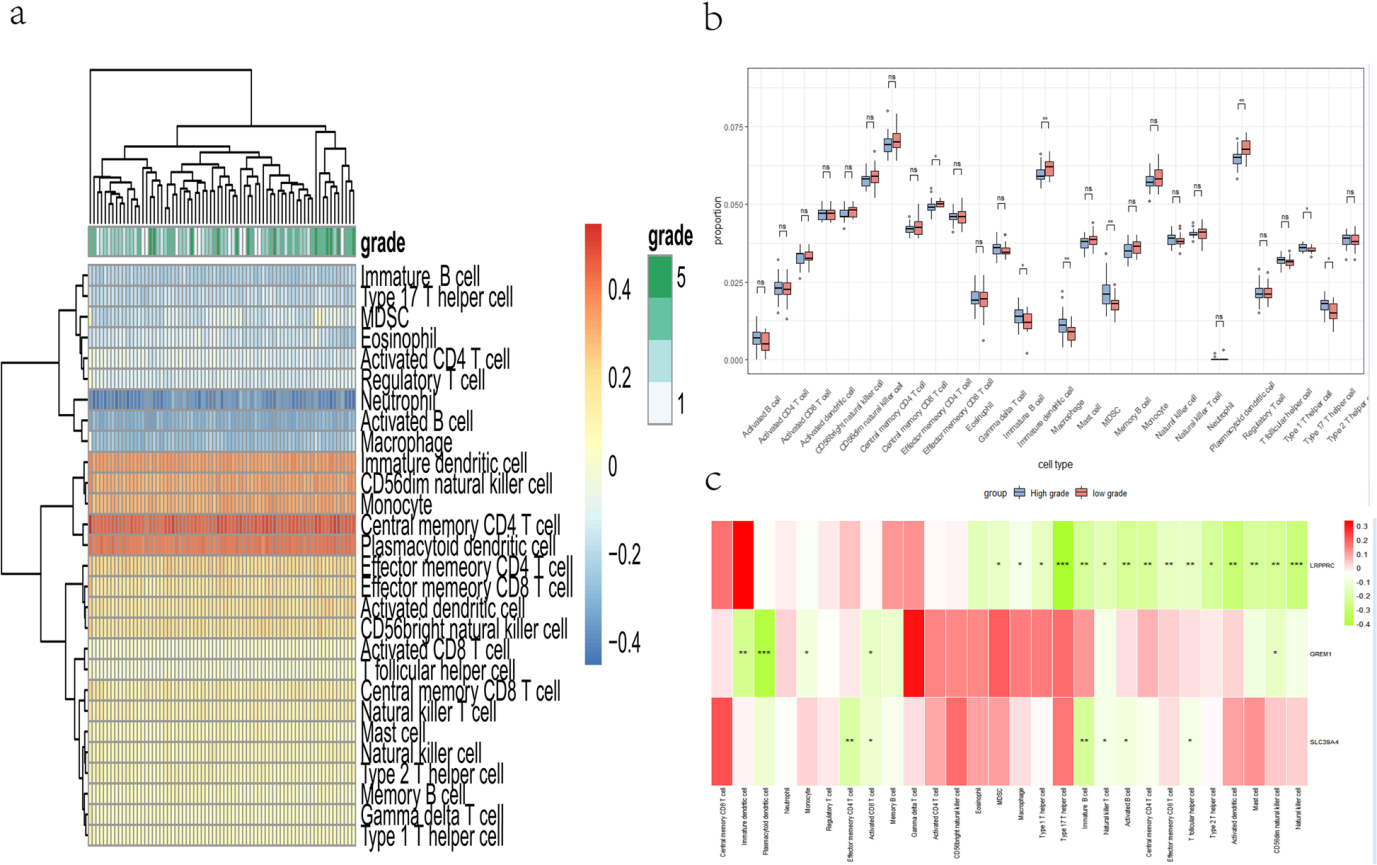
**a** Heatmap result: grades 1 to 5 represent the Thompson grade from I to V and the cool and warm colours symbolise the level of immune cell infiltration. **b** Boxplot: The horizontal axis represents the 28 immune cells from the literature and the vertical axis represents the infiltration fraction of different types of immune cells. **c** Heatmap of the correlation between key genes and immune cells: Green represents a negative correlation, red represents a positive correlation; The number of “*” is shown to indicate the magnitude of the level of statistical significance between groups: “***” means *p*-value < 0.001 “**” means *p*-value < 0.01, “*” means *p*-value < 0.05, “ns” means no significant difference

An infiltration score scale for immune cells was obtained based on the paper (Supplementary material 1 in supplementary materials). The heatmap revealed the level of infiltration of immune cell disc samples with different Thompson grades (Fig. 9a). In order to provide a better contrast between the infiltration of different immune cells in the samples, the samples were grouped into low-level (Thompson grade I-II) and high-level (Thompson grade III-V) and were visualized as box plots (Fig. 9b). The two plots presented here confirmed that the distribution of effector memory CD4 T cell (*p*-value < 0.05), immature B cell (*p*-value < 0.05), type 1 helper T cell (TH1, *p*-value < 0.05), type 17 helper T cell (TH17, *p*-value < 0.05), myeloid-derived suppressor cells (MDSC, *P*-value < 0.01), immature dendritic cell (hiDCs, *p*-value < 0.01), macrophage (*p*-value < 0.01), plasmacytoid dendritic cells (pDC, *p*-value < 0.01) was statistically significantly different between the two groups. Another heatmap also showed the links between our key genes and immune cells (Fig. 9c). The role of key genes in immune infiltration was confirmed. Correlation analysis showed that all 3 key genes were negatively correlated with the proportion of infiltrating immune cells. It can be inferred that the trends of GREM1 with pDC, hiDCs and SLC39A4 with immature B cells are consistent with the development of IVDD.

### Spatial distribution of key genes

**Fig. 10:**
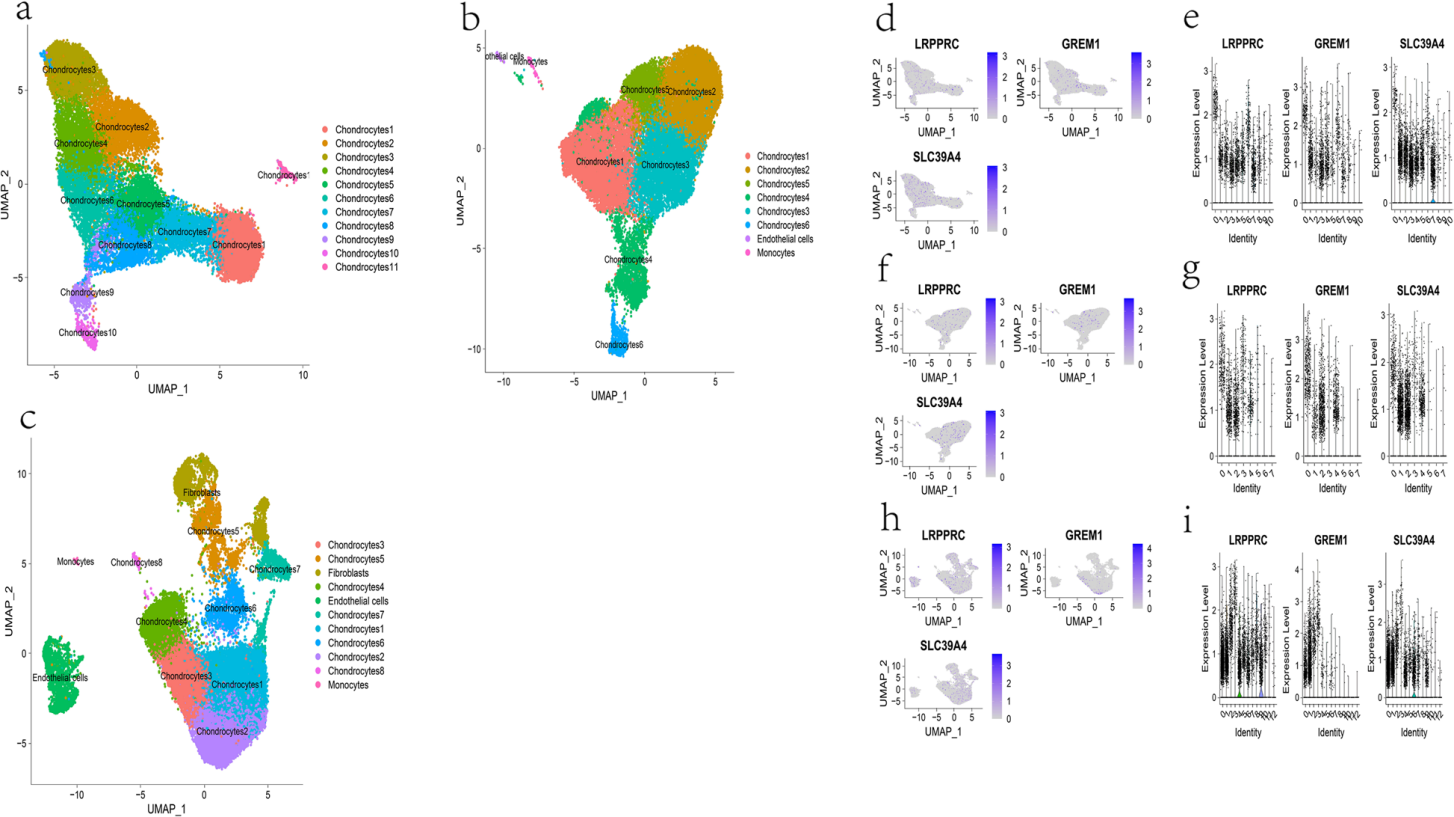
SingleR-based annotation of cell populations of AF (**a**), CEP (**b**) and NP (**c**) containing subpopulations of chondrocytes with different marker genes and some monocytes, stem cells, fibroblasts, and endothelial cells. The UMAP plots and violin plots about the spatial distribution of LRPPRC, GREM1 and SLC39A4 in AF (**d**, **e**), CEP (**f**, **g**) and NP (**h**, **i**). The blue bar depth indicates the counts of genes

A portion of the low-quality cells was filtered as per the expected method and the batch effect was successfully removed from the combined samples in GSE160756 (See supplementary materials 2 and 3 for details about the quality control step). The cell clusterings in the three UMAP maps were automatically annotated according to the “SingleR” package (Fig. 10a, b, c). Accordingly, the spatial expression of LRPPRC, GREM1 and SLC39A4 was visualized in these three tissues (Fig. 10). It can be inferred that these three genes have different distributions within different cell clusters.

**Fig. 11:**
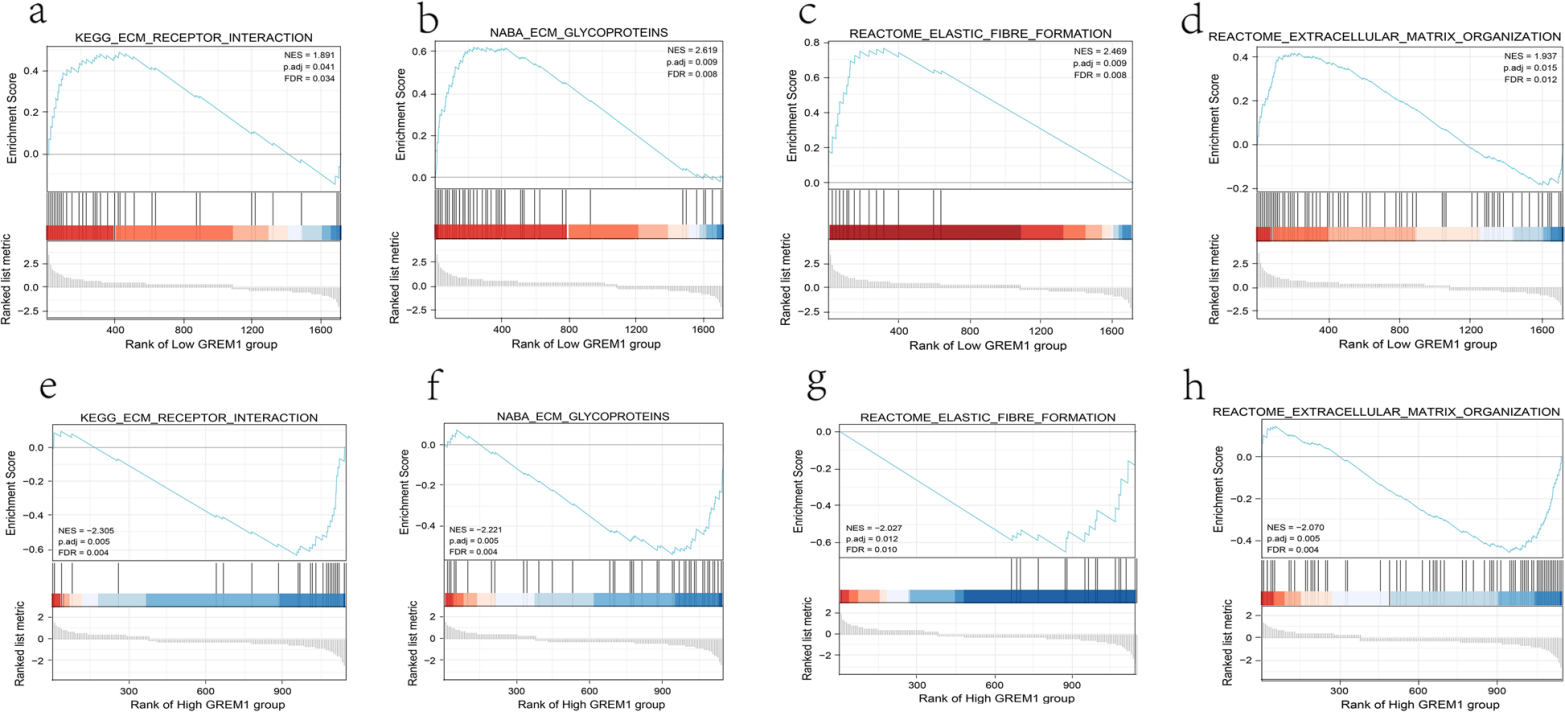
GSEA results for differential genes in “Low GREM1” group (**a**, **b**, **c**, **d**) and “High GREM1” group (**e**, **f**, **g**, **h**)

Using UMAP and violin plots, a significant spatial difference in GREM1 in chondrocytes of NP was found. Based on the information of violin diagram, the chondrocyte clusters were divided into 3 subgroups according to the expression level of GREM1: Chondrocytes 1 to 4 were defined as “High GREM1” group, chondrocytes 5 and 6 were defined as “Medium GREM1” and chondrocytes 7 and 8 were defined as “Low GREM1”. Gene Set Enrichment Analysis (GESA) was used to explore the potential biological functions behind the differential expression of GREM1 in chondrocytes. Several pathways and Gene Ontology annotations that were thought to be associated with the pathological progression of IVDD were selected for GSEA analysis, which included ECM (Extracellular Matrix) receptor interaction, ECM glycoproteins, formation of elastic fibres and organization of ECM [30–33]. The result of GSEA showed that “Low GREM1” group had significantly higher enrichment scores than “High GREM1” group in all of the above gene sets (Fig. 11). Accordingly, it could be assumed that with the increasing of GREM1 expression in chondrocytes, the NP gradually progressed gradual degenerative changes.

**Fig. 12:**
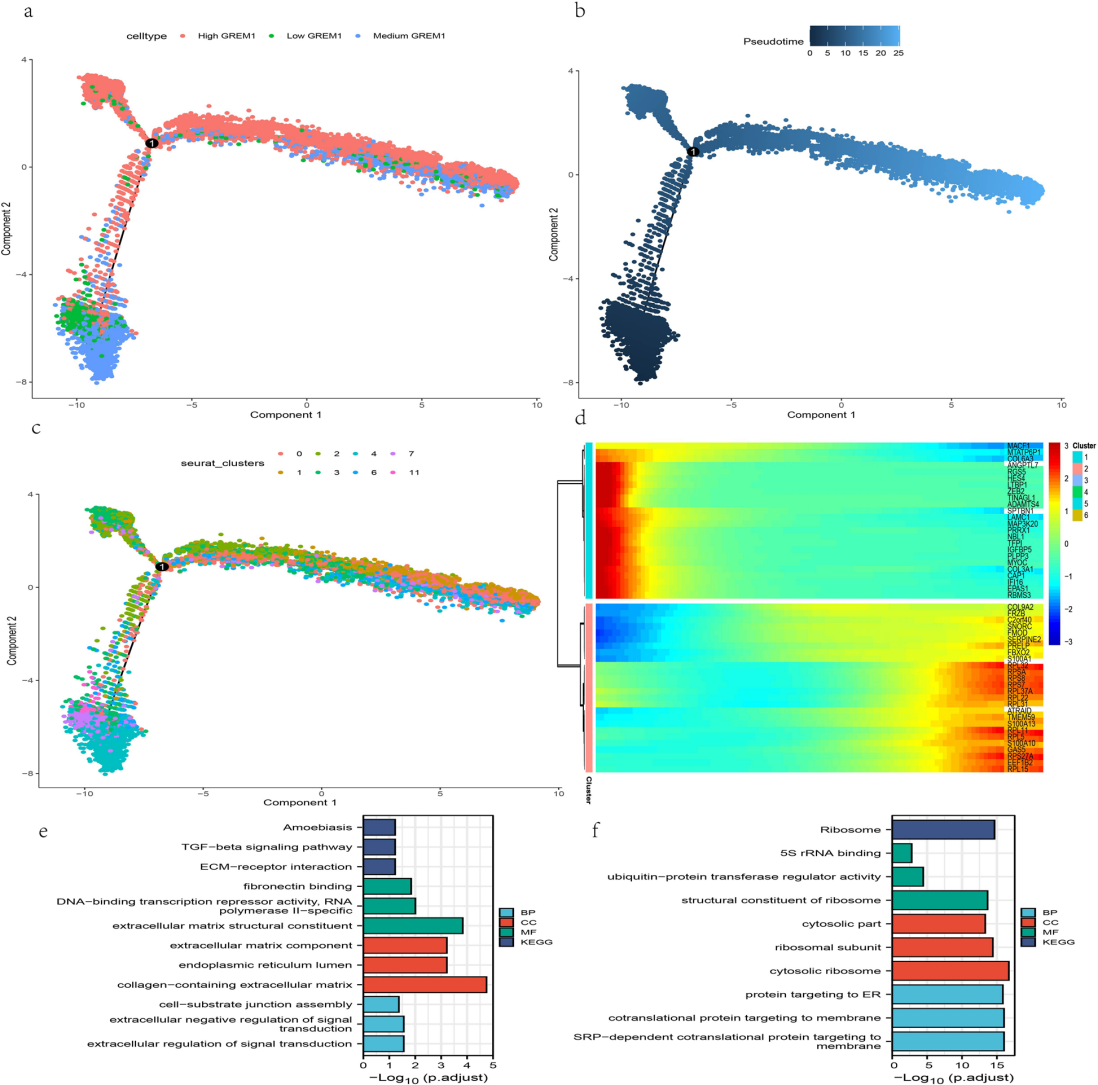
Monocle2-based pseudotime trajectory colored by GREM1 expression subgroup (**a**), pseudotime (**b**) and seurat-based cell clusters (**c**). Pseudotime-related genes heatmap (**d**) and corresponding Go term enrichment results of down-regulated genes (**e**) and up-regulated genes (**f**)

The R package “monocle2” (v.2.18.0) was used to conduct the pseudo-time series analysis between those 3 chondrocyte subgroups (Fig. 12). “Low GREM1” group was defined as the biological starting point for pseudotime based on aforementioned conclusion. The results suggested that the level of GREM1 and the differentiation trajectory of NP chondrocytes were generally consistent. GREM1 may exacerbate the degeneration of NP cells in IVDD. The top 50 genes that had the most significant variation with pseudotime were obtained based on this and plotted the corresponding heat map was plotted (Fig. 10d). Based on the clustering results, the genes can be broadly classified into up-regulated and down-regulated types. Enrichment analysis was conducted for both types of those genes (Fig. 12e, f). It can be seen that down-regulated genes are mainly associated with the Amoebiasis pathway, TGF-β signaling pathway, fibronectin binding etc. In contrast, up-regulated genes are associated with ribosome metabolism, 5S rRNA binding and ubiquitin−protein transferase regulator activity and so on.

## Discussion

IVDD is a prevalent degenerative condition with a complex etiology that presents numerous difficulties for both society and medicine. It is crucial to investigate the underlying processes of IVDD and define it with more precise characteristics that will enable clinical diagnosis and early management [34]. With the progress in research methods, disease markers will be targeted based on sequencing data, leading to the full elucidation of their hidden mechanisms and operational characteristics. 2 independent GEO microarray datasets (GSE34095 and GSE147383) were used for the screening of DEGs and hundreds of DEGs were screened and identified. A large number of relevant entries were enriched using GO enrichment analysis. Among the overlapping GO terms were collagen-containing extracellular matrix, cohesin complex and endosome membrane. The ECM [35, 36] is defined as a complicated macromolecular network composed of collagen, proteoglycan, cohesin and several other glycoproteins that not only plays a supportive, protective and nutritional role for tissue cells, but is also closely related to basic life activities such as cell proliferation, differentiation, metabolism, recognition, adhesion and migration. Degradation of the intervertebral disc ECM is one of the main pathological changes of IVDD in several studies [37, 38]. Yao M et al. [39] used Marein as a protective agent against intervertebral disc ECM degeneration and effectively resisted apoptosis of NP cells. Neidlinger-Wilke C et al. [40] revealed that ECM remodeling altered the mechanical microenvironment of the IVDD thereby compromising disc function. Endosomes are small vesicles produced by cells through endocytosis and this sorting function is essential for critical cellular functions [41]. The biological processes of the majority of DEGs are confirmed by our enrichment results, which also give a theoretical foundation for further investigation.

Previous research frequently exclusively employed the microarray data from the disease group and the control group for simple difference comparison analysis when investigating the genetic biomarkers of diseases. Future bioinformatics studies must delve deeper into statistical findings based on existing clinical and biological theory. 2 other datasets (GSE23130 and GSE70362) were used that included Thompson grades of IVDD to validate the DEGs. Thompson grading system is a common and well-established tool to evaluate degenerative abnormalities on T2-weighted sagittal MRI in daily clinical practice. It differentiates IVDD into 5 levels based on the signal of the central NP and peripheral NF and the height of the intervertebral space. The Thompson grades were included as outcome variables for assessing DEGs. One way that external data can contribute is by preventing false positive or negative results. However, using Thompson grades also increases the applicability of the findings to clinical practice. The idea of variable screening of prediction models to further screen out genes with predictive value was then applied. With fewer variables included, a best subset selection regression was used and our final 3 key genes based on Akaike Information Criterion (AIC) and Bayesian Information Criterion (BIC) were identified. The maximum AUC for prediction models combining these 3 genes can reach 0.78, as shown by the ROC plots, indicating that the key genes in our screen are predictive of the progression of IVDD’s disease course.

The key genes were analyzed based on existing databases such as GeneCards (http://www.genecards.org/). Leucine-rich pentatricopeptide repeat containing (LRPPRC) encodes a kind of leucine-rich protein that has multiple pentatricopeptide repeats (PPR) [42]. The role of this protein is unknown and may play a role in cytoskeletal organization, vesicular transport, or in transcriptional regulation of both nuclear and mitochondrial genes. Various studies demonstrate that high expression of LRPPRC is associated with poor prognosis in a variety of malignancies, such as bladder urothelial carcinoma [43], lung cancer [44] and pancreatic cancer [45]. Maimaiti et al. [46] defined LRPPRC as an N6-methyladenosine (m6A) modification for intracranial aneurysms. Ghavami S et al. [47] reported that LRPPRC was associated with multiple neurodegenerative diseases via autophagy and apoptosis. In our study, LRPPRC is considered to be upregulated in the degenerated disc tissue and expressed in endothelial cells and fibroblasts in addition to different types of chondrocytes. Accordingly, it is speculated that LRPPRC may also be involved in the pathological changes of IVDD in an autophagic or apoptotic pathway, with the involvement of multiple cells.

Gremlin1 (GREM1) is known as a member of the transforming growth factor-β (TGF-β) signaling family encoding antagonists to bone morphogenetic protein (BMP) which potentially plays a role in the regulation of organogenesis, body patterns and tissue differentiation and its related pathways include mainly angiogenesis (CST) and BMP signaling [48, 49]. HKišonaitė M et al. [50] concluded that GREM1 could inhibit BMP2-mediated osteoblast differentiation from in vitro studies. Kobayashi H et al. [51] identified GREM1 and Islr as CAF-specific genes involved in BMP signaling and derived Colorectal Carcinogenesis. Shunlun Chen et al. [52] found that GREM1 promoted myeloid apoptosis and IVDD by inhibiting TGF-β-mediated Smad2/3 phosphorylation, which is coherent with our findings. Although the function of BMP in IVDD is unclear, it is characterized by a variety of and versatility in BMP as well as its signal pathways and inhibitors. Hollenberg AM et al. [53] demonstrated a correlation between the expression level of BMP-2 and Thompson grades, while Haschtmann D et al. [54] concluded that BMP-2 resulted in an up-regulation of Col I and type II, and of aggrecan gene expression. According to most recent studies, BMP may promote AF ossification. The effectiveness of BMP on the overall disc tissue is still unknown, hence it is unclear if it may serve as treatment for IVDD. It was inferred from the prediction models that GREM1 was the strongest predictor among the 3 genes so it will also be the center of our research and discussion afterwards. Our study first proved the potential of GREM1 as a gene marker for IVDD, which is also in line with several previous studies [55, 56]. GREM1 can affect cellular function by multiple mechanisms in addition to acting as a type of BMP inhibitor [57–59]. Whether GREM1 could also promote the development of IVDD is another area that warrants academic attention.

SLC34A4 encodes a member of the zinc/iron-regulated transporter-like protein (ZIP) family which localizes to cell membranes and is required for zinc uptake in the intestine. Metal ion SLC transporters and the Transport of inorganic cations, anions, and amino acids/oligopeptides are two of its related pathways (http://www.genecards.org/). The main diseases associated with SLC39A4 are Acrodermatitis Enteropathica [60], Zinc-Deficiency Type Acrodermatitis [61], and there are relatively few studies other than the aforementioned two conditions. Zinc is a necessary element for humans and is involved in the upkeep of numerous metabolic processes. Recent research has connected metabolic modifications to cell fate. Liang J et al. [62] discovered that type II alveolar epithelial cell ZIP8 deficiency in young mice results in reduced precursor cell function and impaired self-renewal. Chen PH et al. [63] identified an unexpected role for ZIP7 in Ferroptosis by maintaining endoplasmic reticulum homeostasis. These findings may have therapeutic implications for diseases involving iron death and zinc dysregulation. The connection between genes involved in zinc metabolism and IVDD has not been extensively studied. Staszkiewicz R et al. [64] investigated the relationship between the concentration of local metal ions in the patient’s disc tissue and the degree of progression of IVDD. They discovered that the strongest relationships were noted between the concentrations of zinc. It is hypothesized that abnormalities in SLC39A4 cause an imbalance in zinc metabolism inside and outside the disc tissue cells, which may promote IVDD through several pathways, including programmed cell death. Our study suggests that the down-regulation of SLC39A4 may be another significant feature of IVDD. Additional experimental verification is required to determine the precise mechanism and principle.

Current studies on miRNAs in IVDD have confirmed that a variety of miRNAs play critical roles in the process of IVDD through apoptosis, aberrant proliferation, inflammatory response and ECM degradation [65–67]. Another major research interest in miRNA is its important role in exosome therapy [68]. Exosome therapy is achieved by direct in vitro injection of extracellular vehicles (EVs) containing miRNAs or by building vectors to intervene in cellular metabolism using paracrine signaling regulation [69, 70]. However, caution must be exercised when studying the application of miRNAs, as miRNA-mRNA and miRNA-miRNA regulatory pathways may not similar in different tissues [71], and misuse may lead to an imbalance in the ceRNA regulatory network. In this study, meaningful mRNA-miRNA pairs (GREM1-mir-665, LRPPRC-mir-107, LRPPRC/SLC39A4-mir-484, LRPPRC/SLC39A4-miR-103a-3p) were screened for these 3 key genes by combining the miRNA target prediction database miRnet and external miRNA microarray dataset GSE63492. The application and validation of these miRNAs require additional experimental.

The pattern of immune infiltration is another crucial issue. Our findings on the immune infiltration by ssGSEA in patients with various Thompson grades demonstrate a decreased infiltration of effector memory CD4 T cells, pDC, and hiDCs and increased infiltration of TH1, TH17, MDSC, macrophage, and pDCs in disc tissues of high degeneration grade as compared to low degeneration grade. The concomitant association of GREM1, hiDCs, and SLC39A4 with immature B cells is noteworthy. On the one hand, infiltration of immune cells in degenerated discs can further amplify the inflammatory cascade response [72, 73], and on the other hand, immune cells may lead to vascular invasion and the release of neurogenic factors [74]. Our study confirmed the involvement of some immune cells in IVDD and provided potential mechanisms [75, 76]. However, the role of dendritic cells (DCs) in IVDD has not been extensively studied. It is hypothesized that the migration and antigen-presentation functions of DCs are critical for disc tissue inflammation initiation and tolerogenic immune responses [77]. Future experimental investigations must test the aforementioned hypothesis.

There are still many limitations in the current study. For instance, the Thompson grades were demarcated based on our clinical experience which reduced the detail of the outcome variables and was constrained by the sample size. We integrated data from multiple GEO datasets, increasing the sample size on the one hand. However, this might also affect the results due to the heterogeneity of patients or donors (age, gender, location of the intervertebral discs, underlying disease, or other unforeseen biases due to batch effects, etc.). The trend in LRPPRC predicted by logistic regression is the opposite of that screened by the linear model, which might be due to the different definitions of IVDD in different datasets. In the future, the expression of key genes or proteins under different grades should be studied based on Thompson grades. Second, no equivalent degenerated group was included in the study of single-cell transcriptome data. *P*-values were used to screen for significant miRNAs, which could have entailed may result in a degree of false positives. Additionally, the cells were primarily separated into chondrocytes and non-chondrocytes during the single-cell sequencing clustering step. We have performed initial validation of key genes at the RNA and protein level. Our Western Blot results do not confirm the difference in protein levels between LRPPRC and SLC39A4 which may be due to the small sample size and more experiments are still needed in the future. Our future studies will incorporate other omic approaches that incorporate genomics, proteomics and metabolomics, complemented by more experiments, to more fully address the relevant role of IVDD biomarkers.

## Conclusions

In conclusion, 6 DEGs of IVDD were identified by differential analysis of microarray data, and 3 key genes (GREM1, SLC39A4, LRPPRC) were screened by external data validation. The prediction models using four machine learning methods: SVM, RF, XGBoost, and Logistic Regression were validated. Finally, the immune infiltration of key genes was analyzed using the method of ssGSEA and the immune infiltration pattern of IVDD in combination with Thompson grades were predicted. The upstream miRNAs of key genes were predicted using miRNet and external data and the distribution of key genes in NP, AF, CEP was analyzed using single cell sequencing data. The change in GREM1 expression for the distinct transcriptional states was compared using pseudo-time analysis. Our study offers a new perspective to identify credible and effective gene therapy targets in IVDD.

### Supplementary Information

**Additional file 1.** Marker gene dataset for immune cells: “Metagene” represents the name of marker genes. “Cell type” represents different types of immune cells. ” Immunity” represents types of immune response.

**Additional file 2.** Preprocessed AF(a)(b), CEP(c)(d) NP(e)(f) quality control violin plots: The horizontal coordinates indicate different samples, which are simplified here to be expressed as numbers; The vertical coordinates indicate different types of genes:nFeature_RNA and nCount_RNA represent the total number of genes and total number of gene expressions where there is a positive correlation between the two in regular conditions. Percent.mt and percent.ribo indicate the expression ratio of mitochondrial and ribosomal genes. The percentage of mitochondrial genes was controlled to less than 10%.

**Additional file 3.** UMAP and cell clustering maps before and after the removal of the batch effect regarding AF(a)(b),CEP(c)(d) and NP(e)(f): (a)(c)(e) denotes the clustering of samples within the group before removing the batch effect, and it can be seen that there are significant differences in the distribution of different samples; (b)(d)(f) indicates the clustering of cells in each group after removing the batch effect and and performs preliminary cell clustering.

**Additional file 4.**

**Additional file 5.**

## Data Availability

The datasets analysed during the current study are available in Gene Expression Omnibus (GEO) database (http://www.ncbi.nlm.nih.gov/geo/): GSE34095, GSE147383, GSE70632, GSE23130, GSE63492, GSE160756.

## Abbreviations

IVDD: Intervertebral disc degeneration
NP: Nucleus pulposus
AF: Annulus fibrosus
CEP: Cartilaginous endplate
ssGSEA: Single-sample gene set enrichment analysis
ceRNA: Competing endogenous RNAs
DEGs: Differentially expressed genes
PCA: Principal component analysis
GO: Gene Ontology
SVM: Support vector model
RF: RandomForest
XGBoost: Extreme gradient boosting
AUC: Area under curve
ROC: Receiver operator characteristic curve
UMAP: Uniform Manifold Approximation and Projection for Dimension Reduction
LRPPRC: Leucine rich pentatricopeptide repeat containing
GREM1: Gremlin1
SLC39A4: Solute Carrier Family 39 Member 4
BMP: Bone morphogenetic protein
ECM: Extracellular matrix
