# Supplementary material for: GREM1, LRPPRC and SLC39A4 as potential biomarkers of intervertebral disc degeneration: a bioinformatics analysis based on multiple microarray and single-cell sequencing data
Source: BMC Musculoskelet Disord. 2023 Sep 12;24:729. doi: 10.1186/s12891-023-06854-4 (PMC10498557; doi:10.1186/s12891-023-06854-4)
Supplement: Supplementary file 1 — Additional file 1. Marker gene dataset for immune cells: “Metagene” represents the name of marker genes. “Cell type” represents different types of immune cells. ” Immunity” represents types of immune response. [file 12891_2023_6854_MOESM1_ESM.pdf]

Supplementary material 1.pdf: Marker gene dataset for immune cells: “Metagene” represents the name of marker genes. “Cell type” represents different types of immune cells. ” Immunity” represents types of immune response.

Metagene	Cell type	Immunity
ADAM28	Activated B cell	Adaptive
CD180	Activated B cell	Adaptive
CD79B	Activated B cell	Adaptive
BLK	Activated B cell	Adaptive
CD19	Activated B cell	Adaptive
MS4A1	Activated B cell	Adaptive
TNFRSF17	Activated B cell	Adaptive
IGHM	Activated B cell	Adaptive
GNG7	Activated B cell	Adaptive
MICAL3	Activated B cell	Adaptive
SPIB	Activated B cell	Adaptive
HLA-DOB	Activated B cell	Adaptive
IGKC	Activated B cell	Adaptive
PNOC	Activated B cell	Adaptive
FCRL2	Activated B cell	Adaptive
BACH2	Activated B cell	Adaptive
CR2	Activated B cell	Adaptive
TCL1A	Activated B cell	Adaptive
AKNA	Activated B cell	Adaptive
ARHGAP25	Activated B cell	Adaptive
CCL21	Activated B cell	Adaptive
CD27	Activated B cell	Adaptive
CD38	Activated B cell	Adaptive
CLEC17A	Activated B cell	Adaptive
CLEC9A	Activated B cell	Adaptive
CLECL1	Activated B cell	Adaptive
AIM2	Activated CD4 T cell	Adaptive
BIRC3	Activated CD4 T cell	Adaptive
BRIP1	Activated CD4 T cell	Adaptive
CCL20	Activated CD4 T cell	Adaptive
CCL4	Activated CD4 T cell	Adaptive
CCL5	Activated CD4 T cell	Adaptive
CCNB1	Activated CD4 T cell	Adaptive
CCR7	Activated CD4 T cell	Adaptive
DUSP2	Activated CD4 T cell	Adaptive
ESCO2	Activated CD4 T cell	Adaptive
ETS1	Activated CD4 T cell	Adaptive
EXO1	Activated CD4 T cell	Adaptive
EXOC6	Activated CD4 T cell	Adaptive
IARS	Activated CD4 T cell	Adaptive
ITK	Activated CD4 T cell	Adaptive
KIF11	Activated CD4 T cell	Adaptive
KNTC1	Activated CD4 T cell	Adaptive
NUF2	Activated CD4 T cell	Adaptive
PRC1	Activated CD4 T cell	Adaptive
PSAT1	Activated CD4 T cell	Adaptive
RGS1	Activated CD4 T cell	Adaptive
RTKN2	Activated CD4 T cell	Adaptive
SAMSN1	Activated CD4 T cell	Adaptive

SELL	Activated CD4 T cell	Adaptive
TRAT1	Activated CD4 T cell	Adaptive
ADRM1	Activated CD8 T cell	Adaptive
AHSA1	Activated CD8 T cell	Adaptive
C1GALT1C1	Activated CD8 T cell	Adaptive
CCT6B	Activated CD8 T cell	Adaptive
CD37	Activated CD8 T cell	Adaptive
CD3D	Activated CD8 T cell	Adaptive
CD3E	Activated CD8 T cell	Adaptive
CD3G	Activated CD8 T cell	Adaptive
CD69	Activated CD8 T cell	Adaptive
CD8A	Activated CD8 T cell	Adaptive
CETN3	Activated CD8 T cell	Adaptive
CSE1L	Activated CD8 T cell	Adaptive
GEMIN6	Activated CD8 T cell	Adaptive
GNLY	Activated CD8 T cell	Adaptive
GPT2	Activated CD8 T cell	Adaptive
GZMA	Activated CD8 T cell	Adaptive
GZMH	Activated CD8 T cell	Adaptive
GZMK	Activated CD8 T cell	Adaptive
IL2RB	Activated CD8 T cell	Adaptive
LCK	Activated CD8 T cell	Adaptive
MPZL1	Activated CD8 T cell	Adaptive
NKG7	Activated CD8 T cell	Adaptive
PIK3IP1	Activated CD8 T cell	Adaptive
PTRH2	Activated CD8 T cell	Adaptive
TIMM13	Activated CD8 T cell	Adaptive
ZAP70	Activated CD8 T cell	Adaptive
ABHD3	Central memory CD4 T cell	Adaptive
AHNAK	Central memory CD4 T cell	Adaptive
ANXA2P2	Central memory CD4 T cell	Adaptive
AQP3	Central memory CD4 T cell	Adaptive
ATHL1	Central memory CD4 T cell	Adaptive
BMI1	Central memory CD4 T cell	Adaptive
BZW2	Central memory CD4 T cell	Adaptive
CD63	Central memory CD4 T cell	Adaptive
COL4A1	Central memory CD4 T cell	Adaptive
CYLD	Central memory CD4 T cell	Adaptive
ELMO2	Central memory CD4 T cell	Adaptive
FYN	Central memory CD4 T cell	Adaptive
GLIPR1	Central memory CD4 T cell	Adaptive
GSS	Central memory CD4 T cell	Adaptive
IFITM2	Central memory CD4 T cell	Adaptive
ITGB1	Central memory CD4 T cell	Adaptive
ITGB2	Central memory CD4 T cell	Adaptive
KLF5	Central memory CD4 T cell	Adaptive
LSP1	Central memory CD4 T cell	Adaptive
NDUFB9	Central memory CD4 T cell	Adaptive
PKM2	Central memory CD4 T cell	Adaptive
SFXN3	Central memory CD4 T cell	Adaptive

SIRPG	Central memory CD4 T cell	Adaptive
SMAD4	Central memory CD4 T cell	Adaptive
STX4	Central memory CD4 T cell	Adaptive
TRADD	Central memory CD4 T cell	Adaptive
VIM	Central memory CD4 T cell	Adaptive
XRCC6	Central memory CD4 T cell	Adaptive
ACTN4	Central memory CD8 T cell	Adaptive
ADAM12	Central memory CD8 T cell	Adaptive
ADCY9	Central memory CD8 T cell	Adaptive
F13A1	Central memory CD8 T cell	Adaptive
FCER1G	Central memory CD8 T cell	Adaptive
FCGR3B	Central memory CD8 T cell	Adaptive
FGF7	Central memory CD8 T cell	Adaptive
FKBP4	Central memory CD8 T cell	Adaptive
GLUD1	Central memory CD8 T cell	Adaptive
GM2A	Central memory CD8 T cell	Adaptive
GUSB	Central memory CD8 T cell	Adaptive
IL1RN	Central memory CD8 T cell	Adaptive
NOL11	Central memory CD8 T cell	Adaptive
NTRK1	Central memory CD8 T cell	Adaptive
RARA	Central memory CD8 T cell	Adaptive
RNF128	Central memory CD8 T cell	Adaptive
SIGLEC1	Central memory CD8 T cell	Adaptive
TNFRSF11A	Central memory CD8 T cell	Adaptive
TOX4	Central memory CD8 T cell	Adaptive
UBA52	Central memory CD8 T cell	Adaptive
ULBP1	Central memory CD8 T cell	Adaptive
ATM	Effector memeory CD4 T cell	Adaptive
CASP3	Effector memeory CD4 T cell	Adaptive
CASQ1	Effector memeory CD4 T cell	Adaptive
CD300E	Effector memeory CD4 T cell	Adaptive
DARS	Effector memeory CD4 T cell	Adaptive
DOCK9	Effector memeory CD4 T cell	Adaptive
EXOSC9	Effector memeory CD4 T cell	Adaptive
EZH2	Effector memeory CD4 T cell	Adaptive
GDE1	Effector memeory CD4 T cell	Adaptive
IL34	Effector memeory CD4 T cell	Adaptive
NCOA4	Effector memeory CD4 T cell	Adaptive
NEFL	Effector memeory CD4 T cell	Adaptive
PDGFRL	Effector memeory CD4 T cell	Adaptive
PTGS1	Effector memeory CD4 T cell	Adaptive
REPS1	Effector memeory CD4 T cell	Adaptive
SCG2	Effector memeory CD4 T cell	Adaptive
SDPR	Effector memeory CD4 T cell	Adaptive
SIGLEC14	Effector memeory CD4 T cell	Adaptive
SIGLEC6	Effector memeory CD4 T cell	Adaptive
TAL1	Effector memeory CD4 T cell	Adaptive
TFEC	Effector memeory CD4 T cell	Adaptive
TIPIN	Effector memeory CD4 T cell	Adaptive
TPK1	Effector memeory CD4 T cell	Adaptive

UQCRB	Effector memeory CD4 T cell	Adaptive
USP9Y	Effector memeory CD4 T cell	Adaptive
WIPF1	Effector memeory CD4 T cell	Adaptive
ZCRB1	Effector memeory CD4 T cell	Adaptive
ACAP1	Effector memeory CD8 T cell	Adaptive
APOL3	Effector memeory CD8 T cell	Adaptive
ARHGAP10	Effector memeory CD8 T cell	Adaptive
ATP10D	Effector memeory CD8 T cell	Adaptive
C3AR1	Effector memeory CD8 T cell	Adaptive
CCR5	Effector memeory CD8 T cell	Adaptive
CD160	Effector memeory CD8 T cell	Adaptive
CD55	Effector memeory CD8 T cell	Adaptive
CFLAR	Effector memeory CD8 T cell	Adaptive
CMKLR1	Effector memeory CD8 T cell	Adaptive
DAPP1	Effector memeory CD8 T cell	Adaptive
FCRL6	Effector memeory CD8 T cell	Adaptive
FLT3LG	Effector memeory CD8 T cell	Adaptive
GZMM	Effector memeory CD8 T cell	Adaptive
HAPLN3	Effector memeory CD8 T cell	Adaptive
HLA-DMB	Effector memeory CD8 T cell	Adaptive
HLA-DPA1	Effector memeory CD8 T cell	Adaptive
HLA-DPB1	Effector memeory CD8 T cell	Adaptive
IFI16	Effector memeory CD8 T cell	Adaptive
LIME1	Effector memeory CD8 T cell	Adaptive
LTK	Effector memeory CD8 T cell	Adaptive
NFKBIA	Effector memeory CD8 T cell	Adaptive
SETD7	Effector memeory CD8 T cell	Adaptive
SIK1	Effector memeory CD8 T cell	Adaptive
TRIB2	Effector memeory CD8 T cell	Adaptive
ACP5	Gamma delta T cell	Adaptive
AQP9	Gamma delta T cell	Adaptive
BTN3A2	Gamma delta T cell	Adaptive
C1orf54	Gamma delta T cell	Adaptive
CARD8	Gamma delta T cell	Adaptive
CCL18	Gamma delta T cell	Adaptive
CD209	Gamma delta T cell	Adaptive
CD33	Gamma delta T cell	Adaptive
CD36	Gamma delta T cell	Adaptive
CDK5	Gamma delta T cell	Adaptive
IL10RB	Gamma delta T cell	Adaptive
KLRF1	Gamma delta T cell	Adaptive
LGALS1	Gamma delta T cell	Adaptive
MAPK7	Gamma delta T cell	Adaptive
KLHL7	Gamma delta T cell	Adaptive
KRT80	Gamma delta T cell	Adaptive
LAMC1	Gamma delta T cell	Adaptive
LCORL	Gamma delta T cell	Adaptive
LMNB1	Gamma delta T cell	Adaptive
MEIS3P1	Gamma delta T cell	Adaptive
MPL	Gamma delta T cell	Adaptive

FABP1	Gamma delta T cell	Adaptive
FABP5	Gamma delta T cell	Adaptive
FADD	Gamma delta T cell	Adaptive
MFAP3L	Gamma delta T cell	Adaptive
MINPP1	Gamma delta T cell	Adaptive
RPS24	Gamma delta T cell	Adaptive
RPS7	Gamma delta T cell	Adaptive
RPS9	Gamma delta T cell	Adaptive
DBNL	Gamma delta T cell	Adaptive
CCL13	Gamma delta T cell	Adaptive
CD22	Immature B cell	Adaptive
CYBB	Immature B cell	Adaptive
FAM129C	Immature B cell	Adaptive
FCRL1	Immature B cell	Adaptive
FCRL3	Immature B cell	Adaptive
FCRL5	Immature B cell	Adaptive
FCRLA	Immature B cell	Adaptive
HDAC9	Immature B cell	Adaptive
HLA-DQA1	Immature B cell	Adaptive
HVCN1	Immature B cell	Adaptive
KIAA0226	Immature B cell	Adaptive
NCF1	Immature B cell	Adaptive
NCF1B	Immature B cell	Adaptive
P2RY10	Immature B cell	Adaptive
SP100	Immature B cell	Adaptive
TXNIP	Immature B cell	Adaptive
STAP1	Immature B cell	Adaptive
TAGAP	Immature B cell	Adaptive
ZCCHC2	Immature B cell	Adaptive
AICDA	Memory B cell	Adaptive
CCNA2	Memory B cell	Adaptive
CDKN3	Memory B cell	Adaptive
CLCN5	Memory B cell	Adaptive
ENPP1	Memory B cell	Adaptive
FCER1A	Memory B cell	Adaptive
FCRL4	Memory B cell	Adaptive
MYC	Memory B cell	Adaptive
RUNX2	Memory B cell	Adaptive
SORL1	Memory B cell	Adaptive
SOX5	Memory B cell	Adaptive
STAT5A	Memory B cell	Adaptive
STAT5B	Memory B cell	Adaptive
TLR9	Memory B cell	Adaptive
CCL3L1	Regulatory T cell	Adaptive
CD72	Regulatory T cell	Adaptive
CLEC5A	Regulatory T cell	Adaptive
FOXP3	Regulatory T cell	Adaptive
ITGA4	Regulatory T cell	Adaptive
L1CAM	Regulatory T cell	Adaptive
LIPA	Regulatory T cell	Adaptive

LRP1	Regulatory T cell	Adaptive
LRRC42	Regulatory T cell	Adaptive
MARCO	Regulatory T cell	Adaptive
MMP12	Regulatory T cell	Adaptive
MNDA	Regulatory T cell	Adaptive
MRC1	Regulatory T cell	Adaptive
MS4A6A	Regulatory T cell	Adaptive
PELO	Regulatory T cell	Adaptive
PLEK	Regulatory T cell	Adaptive
PRSS23	Regulatory T cell	Adaptive
PTGIR	Regulatory T cell	Adaptive
ST8SIA4	Regulatory T cell	Adaptive
STAB1	Regulatory T cell	Adaptive
B3GAT1	T follicular helper cell	Adaptive
CDK5R1	T follicular helper cell	Adaptive
PDCD1	T follicular helper cell	Adaptive
BCL6	T follicular helper cell	Adaptive
CD200	T follicular helper cell	Adaptive
CD83	T follicular helper cell	Adaptive
CD84	T follicular helper cell	Adaptive
FGF2	T follicular helper cell	Adaptive
GPR18	T follicular helper cell	Adaptive
CEBPA	T follicular helper cell	Adaptive
CECR1	T follicular helper cell	Adaptive
CLEC10A	T follicular helper cell	Adaptive
CLEC4A	T follicular helper cell	Adaptive
CSF1R	T follicular helper cell	Adaptive
CTSS	T follicular helper cell	Adaptive
DMN	T follicular helper cell	Adaptive
DPP4	T follicular helper cell	Adaptive
LRRC32	T follicular helper cell	Adaptive
MC5R	T follicular helper cell	Adaptive
MICA	T follicular helper cell	Adaptive
NCAM1	T follicular helper cell	Adaptive
NCR2	T follicular helper cell	Adaptive
NRP1	T follicular helper cell	Adaptive
PDCD1LG2	T follicular helper cell	Adaptive
PDCD6	T follicular helper cell	Adaptive
PRDX1	T follicular helper cell	Adaptive
RAE1	T follicular helper cell	Adaptive
RAET1E	T follicular helper cell	Adaptive
SIGLEC7	T follicular helper cell	Adaptive
SIGLEC9	T follicular helper cell	Adaptive
TYRO3	T follicular helper cell	Adaptive
CHST12	T follicular helper cell	Adaptive
CLIC3	T follicular helper cell	Adaptive
IVNS1ABP	T follicular helper cell	Adaptive
KIR2DL2	T follicular helper cell	Adaptive
LGMN	T follicular helper cell	Adaptive
CD70	Type 1 T helper cell	Adaptive

TBX21	Type 1 T helper cell	Adaptive
ADAM8	Type 1 T helper cell	Adaptive
AHCYL2	Type 1 T helper cell	Adaptive
ALCAM	Type 1 T helper cell	Adaptive
B3GALNT1	Type 1 T helper cell	Adaptive
BBS12	Type 1 T helper cell	Adaptive
BST1	Type 1 T helper cell	Adaptive
CD151	Type 1 T helper cell	Adaptive
CD47	Type 1 T helper cell	Adaptive
CD48	Type 1 T helper cell	Adaptive
CD52	Type 1 T helper cell	Adaptive
CD53	Type 1 T helper cell	Adaptive
CD59	Type 1 T helper cell	Adaptive
CD6	Type 1 T helper cell	Adaptive
CD68	Type 1 T helper cell	Adaptive
CD7	Type 1 T helper cell	Adaptive
CD96	Type 1 T helper cell	Adaptive
CFHR3	Type 1 T helper cell	Adaptive
CHRM3	Type 1 T helper cell	Adaptive
CLEC7A	Type 1 T helper cell	Adaptive
COL23A1	Type 1 T helper cell	Adaptive
COL4A4	Type 1 T helper cell	Adaptive
COL5A3	Type 1 T helper cell	Adaptive
DAB1	Type 1 T helper cell	Adaptive
DLEU7	Type 1 T helper cell	Adaptive
DOC2B	Type 1 T helper cell	Adaptive
EMP1	Type 1 T helper cell	Adaptive
F12	Type 1 T helper cell	Adaptive
FURIN	Type 1 T helper cell	Adaptive
GAB3	Type 1 T helper cell	Adaptive
GATM	Type 1 T helper cell	Adaptive
GFPT2	Type 1 T helper cell	Adaptive
GPR25	Type 1 T helper cell	Adaptive
GREM2	Type 1 T helper cell	Adaptive
HAVCR1	Type 1 T helper cell	Adaptive
HSD11B1	Type 1 T helper cell	Adaptive
HUNK	Type 1 T helper cell	Adaptive
IGF2	Type 1 T helper cell	Adaptive
RCSD1	Type 1 T helper cell	Adaptive
RYR1	Type 1 T helper cell	Adaptive
SAV1	Type 1 T helper cell	Adaptive
SELE	Type 1 T helper cell	Adaptive
SELP	Type 1 T helper cell	Adaptive
SH3KBP1	Type 1 T helper cell	Adaptive
SIT1	Type 1 T helper cell	Adaptive
SLC35B3	Type 1 T helper cell	Adaptive
SIGLEC10	Type 1 T helper cell	Adaptive
SKAP1	Type 1 T helper cell	Adaptive
THUMPD2	Type 1 T helper cell	Adaptive
TIGIT	Type 1 T helper cell	Adaptive

ZEB2	Type 1 T helper cell	Adaptive
ENC1	Type 1 T helper cell	Adaptive
FAM134B	Type 1 T helper cell	Adaptive
FBXO30	Type 1 T helper cell	Adaptive
FCGR2C	Type 1 T helper cell	Adaptive
STAC	Type 1 T helper cell	Adaptive
LTC4S	Type 1 T helper cell	Adaptive
MAN1B1	Type 1 T helper cell	Adaptive
MDH1	Type 1 T helper cell	Adaptive
MMD	Type 1 T helper cell	Adaptive
RGS16	Type 1 T helper cell	Adaptive
IL12A	Type 1 T helper cell	Adaptive
P2RX5	Type 1 T helper cell	Adaptive
CD97	Type 1 T helper cell	Adaptive
ITGB4	Type 1 T helper cell	Adaptive
ICAM3	Type 1 T helper cell	Adaptive
METRNL	Type 1 T helper cell	Adaptive
TNFRSF1A	Type 1 T helper cell	Adaptive
IRF1	Type 1 T helper cell	Adaptive
HTR2B	Type 1 T helper cell	Adaptive
CALD1	Type 1 T helper cell	Adaptive
MOCOS	Type 1 T helper cell	Adaptive
TRAF3IP2	Type 1 T helper cell	Adaptive
TLR8	Type 1 T helper cell	Adaptive
TRAF1	Type 1 T helper cell	Adaptive
DUSP14	Type 1 T helper cell	Adaptive
IL17A	Type 17 T helper cell	Adaptive
IL17RA	Type 17 T helper cell	Adaptive
C2CD4A	Type 17 T helper cell	Adaptive
C2CD4B	Type 17 T helper cell	Adaptive
CA2	Type 17 T helper cell	Adaptive
CCDC65	Type 17 T helper cell	Adaptive
CEACAM3	Type 17 T helper cell	Adaptive
IL17C	Type 17 T helper cell	Adaptive
IL17F	Type 17 T helper cell	Adaptive
IL17RC	Type 17 T helper cell	Adaptive
IL17RE	Type 17 T helper cell	Adaptive
IL23A	Type 17 T helper cell	Adaptive
ILDR1	Type 17 T helper cell	Adaptive
LONRF3	Type 17 T helper cell	Adaptive
SH2D6	Type 17 T helper cell	Adaptive
TNIP2	Type 17 T helper cell	Adaptive
ABCA1	Type 17 T helper cell	Adaptive
ABCB1	Type 17 T helper cell	Adaptive
ADAMTS12	Type 17 T helper cell	Adaptive
ANK1	Type 17 T helper cell	Adaptive
ANKRD22	Type 17 T helper cell	Adaptive
B3GALT2	Type 17 T helper cell	Adaptive
CAMTA1	Type 17 T helper cell	Adaptive
CCR9	Type 17 T helper cell	Adaptive

CD40	Type 17 T helper cell	Adaptive
GPR44	Type 17 T helper cell	Adaptive
IFT80	Type 17 T helper cell	Adaptive
ASB2	Type 2 T helper cell	Adaptive
CSRP2	Type 2 T helper cell	Adaptive
DAPK1	Type 2 T helper cell	Adaptive
DLC1	Type 2 T helper cell	Adaptive
DNAJC12	Type 2 T helper cell	Adaptive
DUSP6	Type 2 T helper cell	Adaptive
GNAI1	Type 2 T helper cell	Adaptive
LAMP3	Type 2 T helper cell	Adaptive
NRP2	Type 2 T helper cell	Adaptive
OSBPL1A	Type 2 T helper cell	Adaptive
PDE4B	Type 2 T helper cell	Adaptive
PHLDA1	Type 2 T helper cell	Adaptive
PLA2G4A	Type 2 T helper cell	Adaptive
RAB27B	Type 2 T helper cell	Adaptive
RBMS3	Type 2 T helper cell	Adaptive
RNF125	Type 2 T helper cell	Adaptive
TMPRSS3	Type 2 T helper cell	Adaptive
GATA3	Type 2 T helper cell	Adaptive
BIRC5	Type 2 T helper cell	Adaptive
CDC25C	Type 2 T helper cell	Adaptive
CDC7	Type 2 T helper cell	Adaptive
CENPF	Type 2 T helper cell	Adaptive
CXCR6	Type 2 T helper cell	Adaptive
DHFR	Type 2 T helper cell	Adaptive
EVI5	Type 2 T helper cell	Adaptive
GSTA4	Type 2 T helper cell	Adaptive
HELLS	Type 2 T helper cell	Adaptive
IL26	Type 2 T helper cell	Adaptive
LAIR2	Type 2 T helper cell	Adaptive
ABCD1	Activated dendritic cell	Innate
C1QC	Activated dendritic cell	Innate
CAPG	Activated dendritic cell	Innate
CCL3L3	Activated dendritic cell	Innate
CD207	Activated dendritic cell	Innate
CD302	Activated dendritic cell	Innate
ATP5B	Activated dendritic cell	Innate
ATP5L	Activated dendritic cell	Innate
ATP6V1A	Activated dendritic cell	Innate
BCL2L1	Activated dendritic cell	Innate
C1QB	Activated dendritic cell	Innate
SNURF	Activated dendritic cell	Innate
SPCS3	Activated dendritic cell	Innate
CCNA1	Activated dendritic cell	Innate
CEACAM8	Activated dendritic cell	Innate
NOS2	Activated dendritic cell	Innate
SRA1	Activated dendritic cell	Innate
TNFRSF6B	Activated dendritic cell	Innate

TREM1	Activated dendritic cell	Innate
TREML1	Activated dendritic cell	Innate
RHOA	Activated dendritic cell	Innate
SLC25A37	Activated dendritic cell	Innate
TNFSF14	Activated dendritic cell	Innate
TREML4	Activated dendritic cell	Innate
VNN2	Activated dendritic cell	Innate
XPO6	Activated dendritic cell	Innate
CLEC4C	Activated dendritic cell	Innate
TNFAIP2	Activated dendritic cell	Innate
UBD	Activated dendritic cell	Innate
ACTR3	Activated dendritic cell	Innate
RAB1A	Activated dendritic cell	Innate
SLA	Activated dendritic cell	Innate
HLA-DQA2	Activated dendritic cell	Innate
SIGLEC5	Activated dendritic cell	Innate
SLAMF9	Activated dendritic cell	Innate
ABAT	CD56bright natural killer cell	Innate
C11orf75	CD56bright natural killer cell	Innate
C5orf15	CD56bright natural killer cell	Innate
CDHR1	CD56bright natural killer cell	Innate
DCAF12	CD56bright natural killer cell	Innate
DYNLL1	CD56bright natural killer cell	Innate
GPR137B	CD56bright natural killer cell	Innate
HCP5	CD56bright natural killer cell	Innate
HDGFRP2	CD56bright natural killer cell	Innate
KRT86	CD56bright natural killer cell	Innate
MLST8	CD56bright natural killer cell	Innate
ELMOD3	CD56bright natural killer cell	Innate
ENTPD5	CD56bright natural killer cell	Innate
FAM119A	CD56bright natural killer cell	Innate
FAM179A	CD56bright natural killer cell	Innate
CLIC2	CD56bright natural killer cell	Innate
COX7A2L	CD56bright natural killer cell	Innate
CREB3L4	CD56bright natural killer cell	Innate
CSF1	CD56bright natural killer cell	Innate
CSNK2A2	CD56bright natural killer cell	Innate
CSTA	CD56bright natural killer cell	Innate
CSTB	CD56bright natural killer cell	Innate
CTPS	CD56bright natural killer cell	Innate
CTSD	CD56bright natural killer cell	Innate
FST	CD56bright natural killer cell	Innate
GATA2	CD56bright natural killer cell	Innate
GMPR	CD56bright natural killer cell	Innate
HDC	CD56bright natural killer cell	Innate
HEY1	CD56bright natural killer cell	Innate
HOXA1	CD56bright natural killer cell	Innate
HS2ST1	CD56bright natural killer cell	Innate
HS3ST1	CD56bright natural killer cell	Innate
BCL11B	CD56bright natural killer cell	Innate

CDH3	CD56bright natural killer cell	Innate
MYL6B	CD56bright natural killer cell	Innate
NAA16	CD56bright natural killer cell	Innate
ClQA	CD56bright natural killer cell	Innate
ClQB	CD56bright natural killer cell	Innate
CYP27B1	CD56bright natural killer cell	Innate
EIF3M	CD56bright natural killer cell	Innate
CYP27A1	CD56dim natural killer cell	Innate
DDX55	CD56dim natural killer cell	Innate
DYRK2	CD56dim natural killer cell	Innate
RPL37A	CD56dim natural killer cell	Innate
NOTCH3	CD56dim natural killer cell	Innate
AKR7A3	CD56dim natural killer cell	Innate
GPRC5C	CD56dim natural killer cell	Innate
GRIN1	CD56dim natural killer cell	Innate
HLA-E	CD56dim natural killer cell	Innate
PORCN	CD56dim natural killer cell	Innate
PSMC4	CD56dim natural killer cell	Innate
UPP1	CD56dim natural killer cell	Innate
IL21R	CD56dim natural killer cell	Innate
KIR2DS1	CD56dim natural killer cell	Innate
KIR2DS2	CD56dim natural killer cell	Innate
KIR2DS5	CD56dim natural killer cell	Innate
GIPR	Eosinophil	Innate
KRT18P50	Eosinophil	Innate
LRMP	Eosinophil	Innate
FOSB	Eosinophil	Innate
RRP12	Eosinophil	Innate
GPR183	Eosinophil	Innate
NR4A3	Eosinophil	Innate
ST3GAL6	Eosinophil	Innate
DEPDC5	Eosinophil	Innate
PDE6C	Eosinophil	Innate
PKD2L2	Eosinophil	Innate
GPR65	Eosinophil	Innate
IL5RA	Eosinophil	Innate
P2RY14	Eosinophil	Innate
DACH1	Eosinophil	Innate
DAPK2	Eosinophil	Innate
EMR3	Eosinophil	Innate
ACADM	Immature dendritic cell	Innate
AHCYL1	Immature dendritic cell	Innate
ALDH1A2	Immature dendritic cell	Innate
ALDH3A2	Immature dendritic cell	Innate
ALDH9A1	Immature dendritic cell	Innate
ALOX15	Immature dendritic cell	Innate
AMT	Immature dendritic cell	Innate
ARL1	Immature dendritic cell	Innate
ATIC	Immature dendritic cell	Innate
ATP5A1	Immature dendritic cell	Innate

CAPZA1	Immature dendritic cell	Innate
LILRA5	Immature dendritic cell	Innate
RDX	Immature dendritic cell	Innate
RRAGD	Immature dendritic cell	Innate
TACSTD2	Immature dendritic cell	Innate
INPP5F	Immature dendritic cell	Innate
RAB38	Immature dendritic cell	Innate
PLAU	Immature dendritic cell	Innate
CSF3R	Immature dendritic cell	Innate
SLC18A2	Immature dendritic cell	Innate
AMPD2	Immature dendritic cell	Innate
CLTB	Immature dendritic cell	Innate
C1orf162	Immature dendritic cell	Innate
AIF1	Macrophage	Innate
CCL1	Macrophage	Innate
CCL14	Macrophage	Innate
CCL23	Macrophage	Innate
CCL26	Macrophage	Innate
CD300LB	Macrophage	Innate
CNR1	Macrophage	Innate
CNR2	Macrophage	Innate
EIF1	Macrophage	Innate
EIF4A1	Macrophage	Innate
FPR1	Macrophage	Innate
FPR2	Macrophage	Innate
FRAT2	Macrophage	Innate
GPR27	Macrophage	Innate
GPR77	Macrophage	Innate
RNASE2	Macrophage	Innate
MS4A2	Macrophage	Innate
BASP1	Macrophage	Innate
IGSF6	Macrophage	Innate
HK3	Macrophage	Innate
VNN1	Macrophage	Innate
FES	Macrophage	Innate
NPL	Macrophage	Innate
FZD2	Macrophage	Innate
FAM198B	Macrophage	Innate
HNMT	Macrophage	Innate
SLC15A3	Macrophage	Innate
CD4	Macrophage	Innate
TXNDC3	Macrophage	Innate
FRMD4A	Macrophage	Innate
CRYBB1	Macrophage	Innate
HRH1	Macrophage	Innate
WNT5B	Macrophage	Innate
ADAMTS3	Mast cell	Innate
CPA3	Mast cell	Innate
CMA1	Mast cell	Innate
CTSG	Mast cell	Innate

ARHGAP15	Mast cell	Innate
CPM	Mast cell	Innate
FCN1	Mast cell	Innate
FTL	Mast cell	Innate
HSPA6	Mast cell	Innate
ITGA9	Mast cell	Innate
RNASE3	Mast cell	Innate
S100A4	Mast cell	Innate
SIGLEC8	Mast cell	Innate
SLC6A4	Mast cell	Innate
PTGS2	Mast cell	Innate
EGR3	Mast cell	Innate
PILRA	Mast cell	Innate
CCR2	MDSC	Innate
CD14	MDSC	Innate
CD2	MDSC	Innate
CD86	MDSC	Innate
CXCR4	MDSC	Innate
FCGR2A	MDSC	Innate
FCGR2B	MDSC	Innate
FCGR3A	MDSC	Innate
FERMT3	MDSC	Innate
GPSM3	MDSC	Innate
IL18BP	MDSC	Innate
IL4R	MDSC	Innate
ITGAL	MDSC	Innate
ITGAM	MDSC	Innate
PARVG	MDSC	Innate
PSAP	MDSC	Innate
PTGER2	MDSC	Innate
PTGES2	MDSC	Innate
S100A8	MDSC	Innate
S100A9	MDSC	Innate
ASGR2	Monocyte	Innate
CFP	Monocyte	Innate
ASGR1	Monocyte	Innate
CD1D	Monocyte	Innate
UPK3A	Monocyte	Innate
ACTG1	Monocyte	Innate
ANXA5	Monocyte	Innate
ATP6V1B2	Monocyte	Innate
CFL1	Monocyte	Innate
DAZAP2	Monocyte	Innate
CTBS	Monocyte	Innate
EMR4P	Monocyte	Innate
HIVEP2	Monocyte	Innate
MARCKSL1	Monocyte	Innate
MBP	Monocyte	Innate
MMP15	Monocyte	Innate
PNPLA6	Monocyte	Innate

TMBIM6	Monocyte	Innate
PQBP1	Monocyte	Innate
TEX264	Monocyte	Innate
IKZF1	Monocyte	Innate
AKT3	Natural killer cell	Innate
AXL	Natural killer cell	Innate
BST2	Natural killer cell	Innate
CDH2	Natural killer cell	Innate
CRTAM	Natural killer cell	Innate
CSF2RA	Natural killer cell	Innate
CTSZ	Natural killer cell	Innate
CXCL1	Natural killer cell	Innate
CYTH1	Natural killer cell	Innate
DAXX	Natural killer cell	Innate
DGKH	Natural killer cell	Innate
DLL4	Natural killer cell	Innate
DPYD	Natural killer cell	Innate
ERBB3	Natural killer cell	Innate
F11R	Natural killer cell	Innate
FAM27A	Natural killer cell	Innate
FAM49A	Natural killer cell	Innate
FASLG	Natural killer cell	Innate
FCGR1A	Natural killer cell	Innate
FN1	Natural killer cell	Innate
FSTL1	Natural killer cell	Innate
FUCA1	Natural killer cell	Innate
GBP3	Natural killer cell	Innate
GLS2	Natural killer cell	Innate
GRB2	Natural killer cell	Innate
LST1	Natural killer cell	Innate
BCL2	Natural killer cell	Innate
CDC5L	Natural killer cell	Innate
FGF18	Natural killer cell	Innate
FUT5	Natural killer cell	Innate
FZR1	Natural killer cell	Innate
GAGE2	Natural killer cell	Innate
IGFBP5	Natural killer cell	Innate
KANK2	Natural killer cell	Innate
LDB3	Natural killer cell	Innate
BTN2A2	Natural killer T cell	Innate
CD101	Natural killer T cell	Innate
CD109	Natural killer T cell	Innate
CNPY3	Natural killer T cell	Innate
CNPY4	Natural killer T cell	Innate
CREB1	Natural killer T cell	Innate
CRTC2	Natural killer T cell	Innate
CRTC3	Natural killer T cell	Innate
CSF2	Natural killer T cell	Innate
KLRC1	Natural killer T cell	Innate
FUT4	Natural killer T cell	Innate

ICAM2	Natural killer T cell	Innate
IL32	Natural killer T cell	Innate
LAMP2	Natural killer T cell	Innate
LILRB5	Natural killer T cell	Innate
KLRG1	Natural killer T cell	Innate
HSPA4	Natural killer T cell	Innate
HSPB6	Natural killer T cell	Innate
ISM2	Natural killer T cell	Innate
ITIH2	Natural killer T cell	Innate
KDM4C	Natural killer T cell	Innate
KIR2DS4	Natural killer T cell	Innate
KIRREL3	Natural killer T cell	Innate
SDCBP	Natural killer T cell	Innate
NFATC2IP	Natural killer T cell	Innate
MICB	Natural killer T cell	Innate
KIR2DL1	Natural killer T cell	Innate
KIR2DL3	Natural killer T cell	Innate
KIR3DL1	Natural killer T cell	Innate
KIR3DL2	Natural killer T cell	Innate
NCR1	Natural killer T cell	Innate
FOSL1	Natural killer T cell	Innate
TSLP	Natural killer T cell	Innate
SLC7A7	Natural killer T cell	Innate
SPP1	Natural killer T cell	Innate
TREM2	Natural killer T cell	Innate
UBASH3A	Natural killer T cell	Innate
YBX2	Natural killer T cell	Innate
CCDC88A	Natural killer T cell	Innate
CLEC1A	Natural killer T cell	Innate
THBD	Natural killer T cell	Innate
PDPN	Natural killer T cell	Innate
VCAM1	Natural killer T cell	Innate
EMR1	Natural killer T cell	Innate
CREB5	Neutrophil	Innate
CDA	Neutrophil	Innate
CHST15	Neutrophil	Innate
S100A12	Neutrophil	Innate
APOBEC3A	Neutrophil	Innate
CASP5	Neutrophil	Innate
MMP25	Neutrophil	Innate
HAL	Neutrophil	Innate
C1orf183	Neutrophil	Innate
FFAR2	Neutrophil	Innate
MAK	Neutrophil	Innate
CXCR1	Neutrophil	Innate
STEAP4	Neutrophil	Innate
MGAM	Neutrophil	Innate
BTNL8	Neutrophil	Innate
CXCR2	Neutrophil	Innate
TNFRSF10C	Neutrophil	Innate

VNN3	Neutrophil	Innate
CBX6	Plasmacytoid dendritic cell	Innate
DAB2	Plasmacytoid dendritic cell	Innate
DDX17	Plasmacytoid dendritic cell	Innate
HIGD1A	Plasmacytoid dendritic cell	Innate
IDH3A	Plasmacytoid dendritic cell	Innate
IL3RA	Plasmacytoid dendritic cell	Innate
MAGED1	Plasmacytoid dendritic cell	Innate
NUCB2	Plasmacytoid dendritic cell	Innate
OFD1	Plasmacytoid dendritic cell	Innate
OGT	Plasmacytoid dendritic cell	Innate
PDIA4	Plasmacytoid dendritic cell	Innate
SERTAD2	Plasmacytoid dendritic cell	Innate
SIRPA	Plasmacytoid dendritic cell	Innate
TMED2	Plasmacytoid dendritic cell	Innate
ENG	Plasmacytoid dendritic cell	Innate
FCAR	Plasmacytoid dendritic cell	Innate
IGF1	Plasmacytoid dendritic cell	Innate
ITGA2B	Plasmacytoid dendritic cell	Innate
GABARAP	Plasmacytoid dendritic cell	Innate
GPX1	Plasmacytoid dendritic cell	Innate
KRT23	Plasmacytoid dendritic cell	Innate
PROK2	Plasmacytoid dendritic cell	Innate
RALB	Plasmacytoid dendritic cell	Innate
RETNLB	Plasmacytoid dendritic cell	Innate
RNF141	Plasmacytoid dendritic cell	Innate
SEC14L1	Plasmacytoid dendritic cell	Innate
SEPX1	Plasmacytoid dendritic cell	Innate
EMP3	Plasmacytoid dendritic cell	Innate
CD300LF	Plasmacytoid dendritic cell	Innate
ABTB1	Plasmacytoid dendritic cell	Innate
KLHL21	Plasmacytoid dendritic cell	Innate
PHRF1	Plasmacytoid dendritic cell	Innate
