# Supplementary material for: GREM1, LRPPRC and SLC39A4 as potential biomarkers of intervertebral disc degeneration: a bioinformatics analysis based on multiple microarray and single-cell sequencing data
Source: BMC Musculoskelet Disord. 2023 Sep 12;24:729. doi: 10.1186/s12891-023-06854-4 (PMC10498557; doi:10.1186/s12891-023-06854-4)

Supplementary material 2.pdf: Preprocessed AF(a)(b), CEP(c)(d) NP(e)(f) quality control violin plots: The horizontal coordinates indicate different samples, which are simplified here to be expressed as numbers;The vertical coordinates indicate different types of genes:nFeature_RNA and nCount_RNA represent the total number of genes and total number of gene expressions where there is a positive correlation between the two in regular conditions. Percent.mt and percent.ribo indicate the expression ratio of mitochondrial and ribosomal genes. The percentage of mitochondrial genes was controlled to less than 10%.

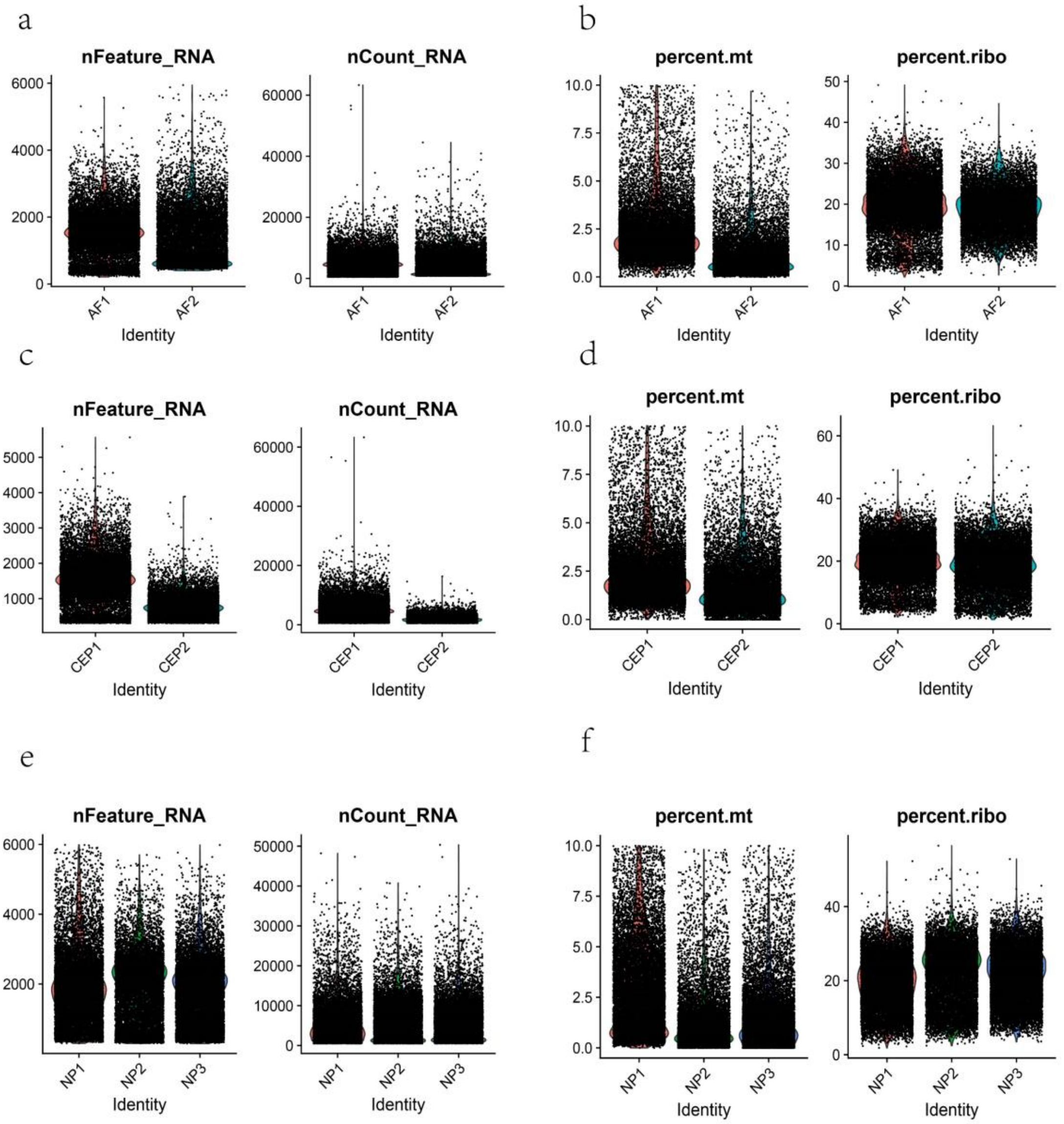

Supplement: Supplementary file 2 — Additional file 2. Preprocessed AF(a)(b), CEP(c)(d) NP(e)(f) quality control violin plots: The horizontal coordinates indicate different samples, which are simplified here to be expressed as numbers; The vertical coordinates indicate different types of genes:nFeature_RNA and nCount_RNA represent the total number of genes and total number of gene expressions where there is a positive correlation between the two in regular conditions. Percent.mt and percent.ribo indicate the expression ratio of mitochondrial and ribosomal genes. The percentage of mitochondrial genes was controlled to less than 10%. [file 12891_2023_6854_MOESM2_ESM.pdf]
