# Supplementary material for: GREM1, LRPPRC and SLC39A4 as potential biomarkers of intervertebral disc degeneration: a bioinformatics analysis based on multiple microarray and single-cell sequencing data
Source: BMC Musculoskelet Disord. 2023 Sep 12;24:729. doi: 10.1186/s12891-023-06854-4 (PMC10498557; doi:10.1186/s12891-023-06854-4)

Supplementary material 3.pdf: UMAP and cell clustering maps before and after the removal of the batch effect regarding AF(a)(b),CEP(c)(d) and NP(e)(f) :(a)(c)(e) denotes the clustering of samples within the group before removing the batch effect, and it can be seen that there are significant differences in the distribution of different samples; (b)(d)(f) indicates the clustering of cells in each group after removing the batch effect and and performs preliminary cell clustering.

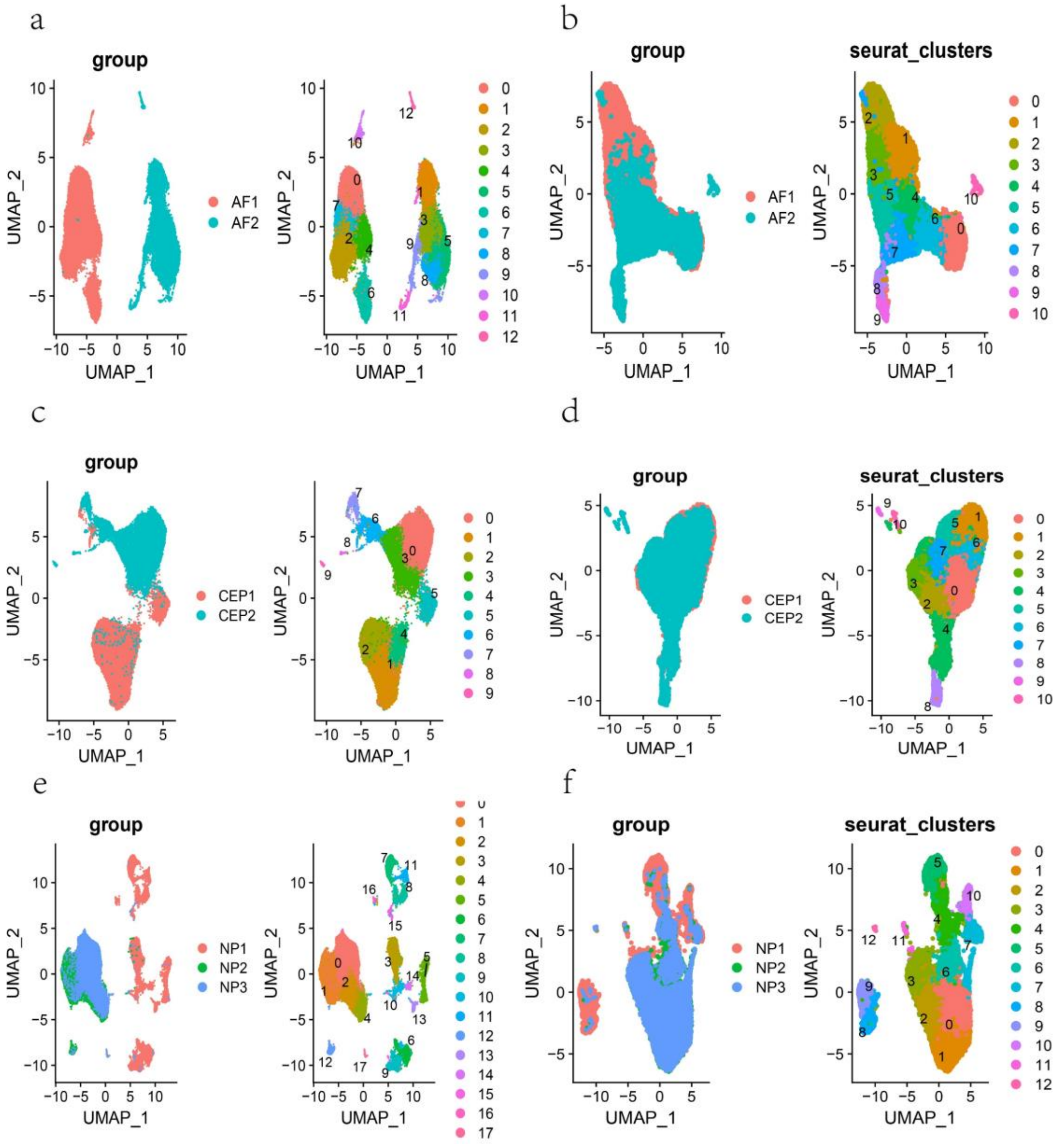

Supplement: Supplementary file 3 — Additional file 3. UMAP and cell clustering maps before and after the removal of the batch effect regarding AF(a)(b),CEP(c)(d) and NP(e)(f): (a)(c)(e) denotes the clustering of samples within the group before removing the batch effect, and it can be seen that there are significant differences in the distribution of different samples; (b)(d)(f) indicates the clustering of cells in each group after removing the batch effect and and performs preliminary cell clustering. [file 12891_2023_6854_MOESM3_ESM.pdf]
