# Supplementary figures and images for: GREM1, LRPPRC and SLC39A4 as potential biomarkers of intervertebral disc degeneration: a bioinformatics analysis based on multiple microarray and single-cell sequencing data
Source: BMC Musculoskelet Disord. 2023 Sep 12;24:729. doi: 10.1186/s12891-023-06854-4 (PMC10498557; doi:10.1186/s12891-023-06854-4)

GAPDH

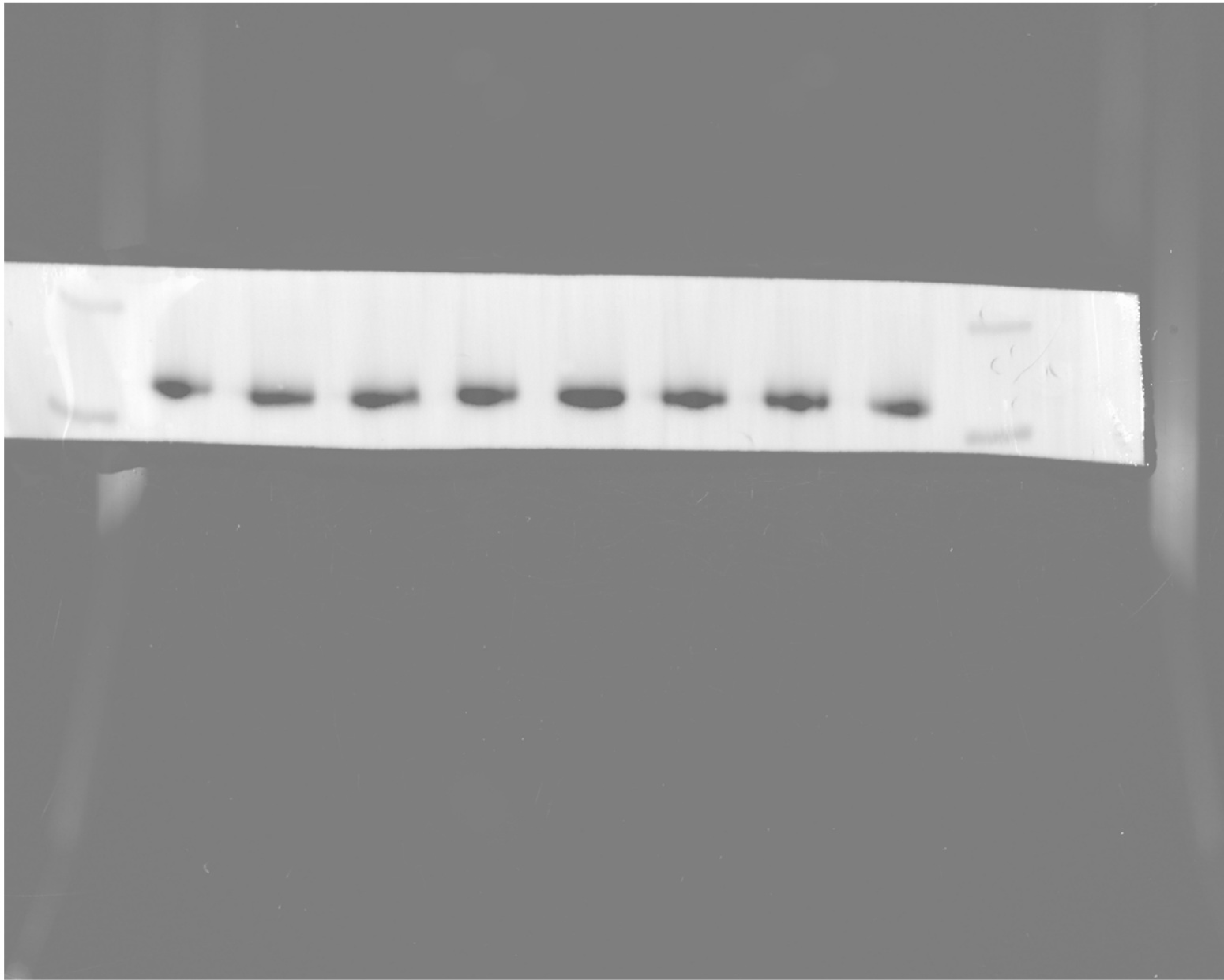

45KDa  
35KDa

GREM1

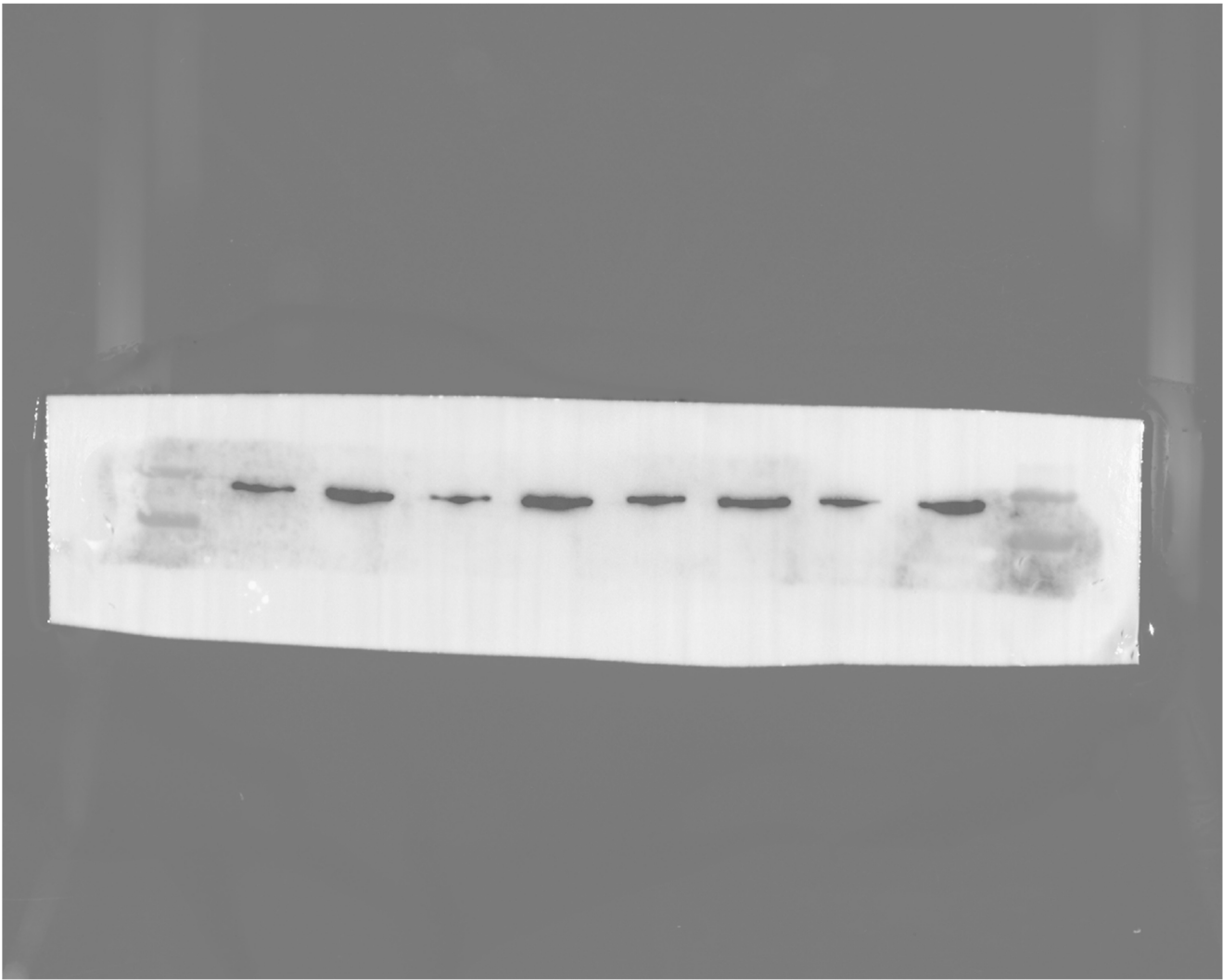

35KDa  
25KDa

LRPPRC

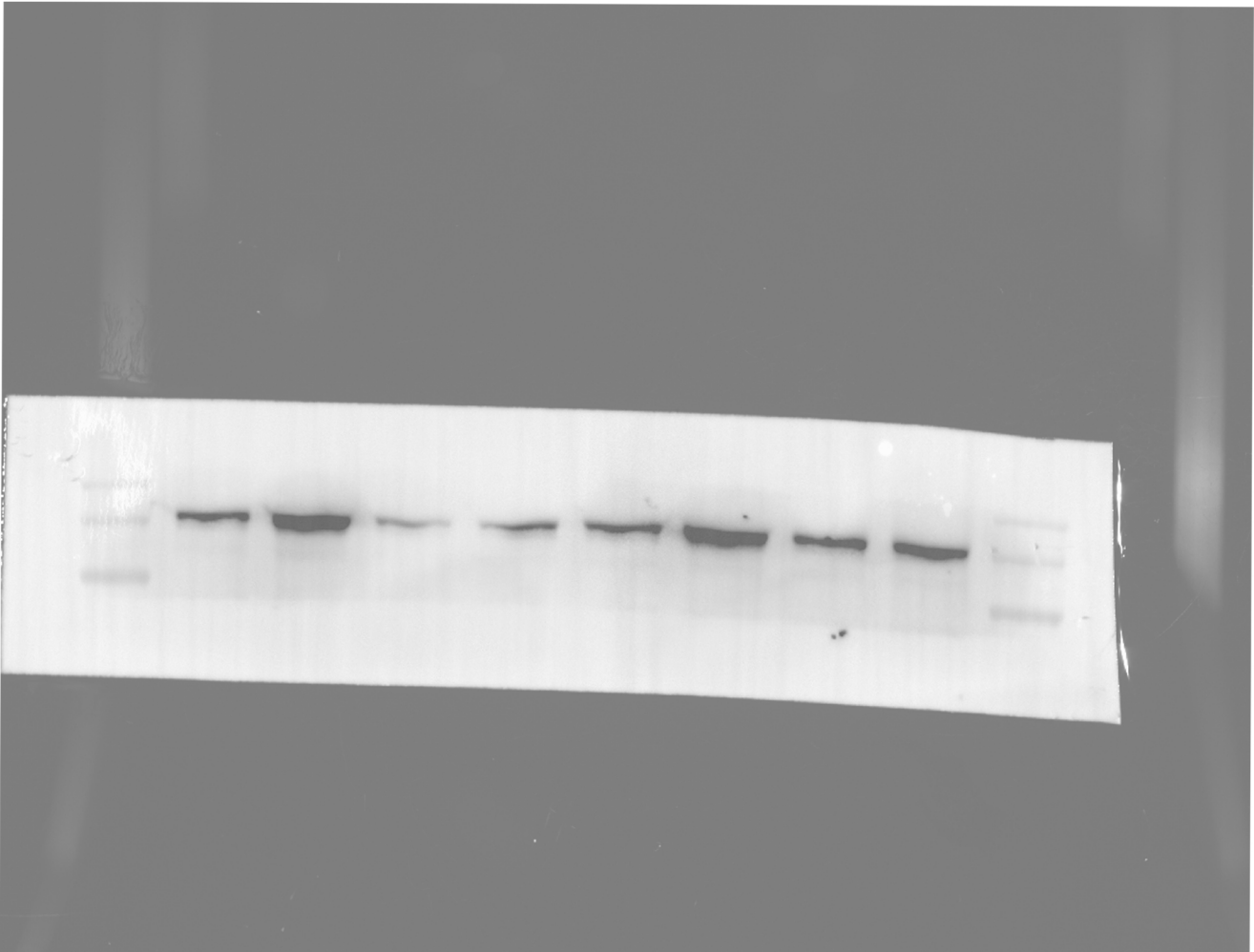

180KDa  
135KDa  
100KDa

SLC39A4(ZIP4)

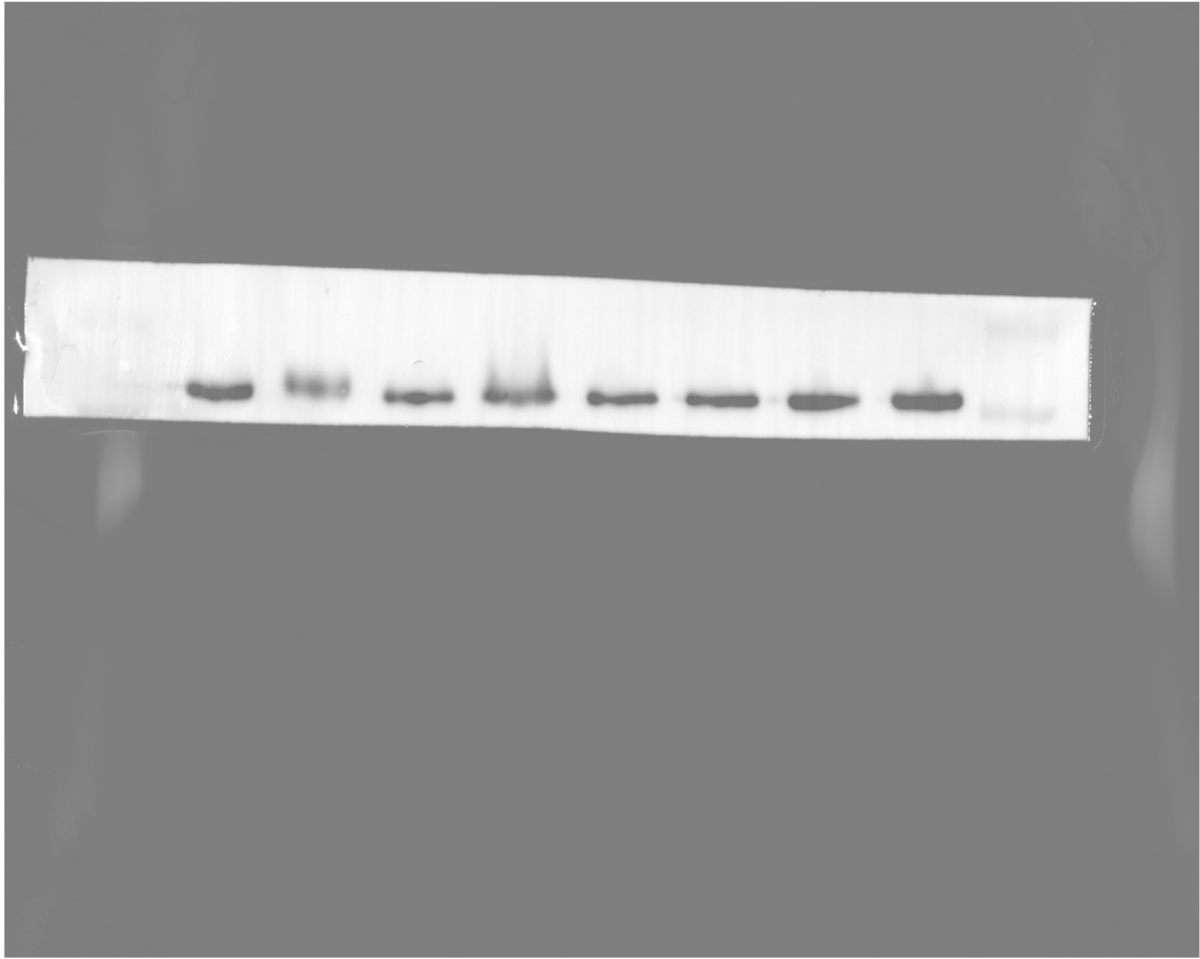

75KDa  
65KDa

Supplement: Supplementary file 4 — Additional file 4. [file 12891_2023_6854_MOESM4_ESM.pdf]
