# Supplementary material for: GREM1, LRPPRC and SLC39A4 as potential biomarkers of intervertebral disc degeneration: a bioinformatics analysis based on multiple microarray and single-cell sequencing data
Source: BMC Musculoskelet Disord. 2023 Sep 12;24:729. doi: 10.1186/s12891-023-06854-4 (PMC10498557; doi:10.1186/s12891-023-06854-4)
Supplement: Supplementary file 5 — Additional file 5. [file 12891_2023_6854_MOESM5_ESM.docx]

|  | Accession | Age | Gender | Grade | Other information |
| --- | --- | --- | --- | --- | --- |
| GSE147383 | GSM4429807 | 18 | male | - | Control |
|  | GSM4429808 | 18 | male | - | Control |
|  | GSM4429809 | 16 | female | - | Control |
|  | GSM4429810 | 16 | female | - | Control |
|  | GSM4429811 | 63 | female | - | Degenerated |
|  | GSM4429812 | 63 | female | - | Degenerated |
|  | GSM4429813 | 48 | male | - | Degenerated |
|  | GSM4429814 | 48 | male | - | Degenerated |
| GSE34095 | GSM841717 | 11 | - | - | Control |
|  | GSM841718 | 49 | - | - | Degenerated |
|  | GSM841719 | 16 | - | - | Control |
|  | GSM841720 | 54 | - | - | Degenerated |
|  | GSM841721 | 15 | - | - | Control |
|  | GSM841722 | 51 | - | **-** | Degenerated |
| GSE70362 | GSM1725780 | - | - | III | - |
|  | GSM1725781 | - | - | III | - |
|  | GSM1725782 | - | - | IV | - |
|  | GSM1725783 | - | - | IV | - |
|  | GSM1725784 | - | - | III | - |
|  | GSM1725785 | - | - | III | - |
|  | GSM1725786 | - | - | IV | - |
|  | GSM1725787 | - | - | IV | - |
|  | GSM1725788 | - | - | IV | - |
|  | GSM1725789 | - | - | IV | - |
|  | GSM1725790 | - | - | V | - |
|  | GSM1725791 | - | - | V | - |
|  | GSM1725792 | - | - | IV | - |
|  | GSM1725793 | - | - | IV | - |
|  | GSM1725794 | - | - | V | - |
|  | GSM1725795 | - | - | V | - |
|  | GSM1725796 | - | - | III | - |
|  | GSM1725797 | - | - | III | - |
|  | GSM1725798 | - | - | IV | - |
|  | GSM1725799 | - | - | IV | - |
|  | GSM1725800 | - | - | I | - |
|  | GSM1725801 | - | - | I | - |
|  | GSM1725802 | - | - | II | - |
|  | GSM1725803 | - | - | II | - |
|  | GSM1725804 | - | - | V | - |
|  | GSM1725805 | - | - | V | - |
|  | GSM1725806 | - | - | II | - |
|  | GSM1725807 | - | - | II | - |
|  | GSM1725808 | - | - | V | - |
|  | GSM1725809 | - | - | V | - |
|  | GSM1725810 | - | - | I | - |
|  | GSM1725811 | - | - | I | - |
|  | GSM1725812 | - | - | I-II | - |
|  | GSM1725813 | - | - | I-II | - |
|  | GSM1725814 | - | - | I | - |
|  | GSM1725815 | - | - | I | - |
|  | GSM1725816 | - | - | III | - |
|  | GSM1725817 | - | - | III | - |
|  | GSM1725818 | - | - | IV | - |
|  | GSM1725819 | - | - | IV | - |
|  | GSM1725820 | - | - | III | - |
|  | GSM1725821 | - | - | III | - |
|  | GSM1725822 | - | - | I | - |
|  | GSM1725823 | - | - | I | - |
|  | GSM1725824 | - | - | III | - |
|  | GSM1725825 | - | - | III | - |
|  | GSM1725826 | - | - | I-II | - |
|  | GSM1725827 | - | - | I-II | - |
| GSE23130 | GSM569830 | - | - | III | - |
|  | GSM569831 | - | - | IV | - |
|  | GSM569832 | - | - | III | - |
|  | GSM569833 | - | - | II | - |
|  | GSM569834 | - | - | IV | - |
|  | GSM569835 | - | - | III | - |
|  | GSM569836 | - | - | III | - |
|  | GSM569837 | - | - | III | - |
|  | GSM569838 | - | - | III | - |
|  | GSM569839 | - | - | II | - |
|  | GSM569840 | - | - | II | - |
|  | GSM569841 | - | - | II | - |
|  | GSM569842 | - | - | I | - |
|  | GSM569843 | - | - | IV | - |
|  | GSM569844 | - | - | III | - |
|  | GSM569845 | - | - | III | - |
|  | GSM569846 | - | - | III | - |
|  | GSM569847 | - | - | II | - |
|  | GSM569848 | - | - | IV | - |
|  | GSM569849 | - | - | IV | - |
|  | GSM569850 | - | - | V | - |
|  | GSM569851 | - | - | V | - |
|  | GSM569852 | - | - | V | - |
| GSE63492 | GSM1551024 | 33 | male | I | Control |
|  | GSM1551025 | 35 | male | I | Control |
|  | GSM1551026 | 41 | male | I | Control |
|  | GSM1551027 | 43 | female | I | Control |
|  | GSM1551028 | 52 | male | I | Control |
|  | GSM1551029 | 32 | female | V | Degenerated |
|  | GSM1551030 | 38 | male | V | Degenerated |
|  | GSM1551031 | 42 | male | IV | Degenerated |
|  | GSM1551032 | 45 | male | V | Degenerated |
|  | GSM1551033 | 27 | female | V | Degenerated |
| GSE160756 | - | - | - | - | Single-cell |

Basic information about the datasets from GEO database:

Accession: Sample names from GEO database

Grade: Thompson Grades level
